# Levels of metals and persistent organic pollutants in traditional foods consumed by First Nations living on-reserve in Canada

**DOI:** 10.17269/s41997-021-00495-7

**Published:** 2021-06-28

**Authors:** Hing Man Chan, Kavita Singh, Malek Batal, Lesya Marushka, Constantine Tikhonov, Tonio Sadik, Harold Schwartz, Amy Ing, Karen Fediuk

**Affiliations:** 1 Department of Biology, University of Ottawa, 30 Marie Curie, Ottawa, ON K1N 6N5 Canada; 2 Département de Nutrition, Faculté de Médecine, Pavillon Liliane de Stewart, Université de Montréal, C.P. 6128, succ. Centre-Ville, Montréal, QC H3T 1A8 Canada; 3 Centre de recherche en santé publique de l’Université de Montréal et du CIUSS du Centre-sud-de-l’Île-de-Montréal (CReSP), 7101 avenue du Parc, Montréal, QC H3N 1X7 Canada; 4 First Nations and Inuit Health Branch, Indigenous Services Canada, Ottawa, ON Canada; 5 Assembly of First Nations, 55 Metcalfe Street, Suite 1600, Ottawa, ON K1P 6L5 Canada

**Keywords:** First Nations, Canada, Traditional foods, Metals, Persistent organic pollutants, Premières Nations, Canada, aliments traditionnels, métaux, polluants organiques persistants

## Abstract

**Objectives:**

First Nations may have a higher risk of contaminant exposure from the consumption of traditional foods. The objective of this study was to measure concentrations of metals and organochlorines in traditional foods commonly consumed by First Nations in Canada and estimate the risk from dietary exposure.

**Methods:**

Data were collected from the participatory First Nations Food, Nutrition and Environment Study (2008–2018). Traditional food samples were collected by community members and concentrations of metals and organochlorines were measured. The population-weighted mean daily contaminant intake from traditional food items was estimated. Hazard quotients (HQs) were calculated by dividing contaminant intake with the toxicological reference values (TRVs).

**Results:**

A total of 2061 food samples (different parts and organs) from 221 species were collected. The highest concentrations of cadmium were found in the kidneys of land mammals: moose kidney was the most significant contributor to intake. The meat of land mammals and birds had the highest lead concentrations and were the most significant contributors to intake. Arsenic was highest in seaweed, and prawn was the most significant contributor. Mercury and methyl mercury were highest in harp seal meat, with walleye/pickerel contributing most to intake. Harp seal meat also had the highest p,p′-DDE and PCB concentrations, and ooligan grease and salmon were the most significant contributors to intake. The percentage of adults eating traditional food who exceeded the TRVs was 1.9% for cadmium, 3.7% for lead, 13.6% for arsenic, 0.7% for mercury, and 0% for p,p′-DDE and PCBs. All median HQs, and most 95th percentile HQs, were less than 1.

**Conclusion:**

These results can be used as a baseline of contaminant levels and exposure in traditional foods for future monitoring programs and to support risk assessment programs.

**Supplementary Information:**

The online version contains supplementary material available at 10.17269/s41997-021-00495-7.

## Résumé

**Objectifs:**

Les Premières Nations peuvent avoir un risque plus élevé d’exposition aux contaminants en raison de la consommation d’aliments traditionnels. L’objectif de cette étude était de mesurer les concentrations de métaux et d’organochlorés dans les aliments traditionnels couramment consommés par les Premières Nations au Canada et estimer le risque d’exposition alimentaire.

**Méthodes:**

Les données ont été recueillies dans le cadre de l’Étude sur l’alimentation, la nutrition et l’environnement des Premières Nations (2008–2018), une étude participative. Des échantillons d’aliments traditionnels ont été prélevés par les membres de la communauté et les concentrations de métaux et d’organochlorés ont été mesurées. L’apport quotidien moyen pondéré pour la population de contaminants provenant du système alimentaire traditionnel a été estimé. Les quotients de risque (QR) ont été calculés en divisant l’apport par les valeurs toxicologiques de référence (VTR).

**Résultats:**

Un total de 2 061 échantillons d’aliments (différentes parties et organes) de 221 espèces ont été collectés. Les concentrations les plus élevées de cadmium ont été trouvées dans les reins des mammifères terrestres : le rein d’orignal était le principal contributeur aux apports en cadmium. La viande de mammifères terrestres et d’oiseaux présentait les concentrations de plomb les plus élevées et était le principal contributeur aux apports en plomb. La concentration en arsenic était la plus élevée dans les algues tandis que la crevette était le contributeur le plus important pour ce contaminant. Les concentrations de mercure et de méthylmercure étaient les plus élevées dans la viande de phoque du Groenland, le doré jaune et le brochet contribuant le plus aux apports de ces contaminants. La viande de phoque du Groenland présentait également les concentrations les plus élevées de p,p′-DDE et de BPC, et la graisse d’eulikan et le saumon en étaient les principaux contributeurs. Le pourcentage d’adultes consommant des aliments traditionnels qui dépassaient les VTR était de 1,9 % pour le cadmium, 3,7 % pour le plomb, 13,6 % pour l’arsenic, 0,7 % pour le mercure et 0 % pour le p,p′-DDE et les PCB. Tous les QR médians et la plupart des QR du 95e centile étaient inférieurs à 1.

**Conclusion:**

Ces résultats peuvent être utilisés comme référence pour les futurs programmes de surveillance et pour soutenir les programmes d’évaluation des risques.

## Introduction

The traditional food systems of First Nations confer multiple cultural and nutritional benefits, which are not replaceable with market-based items. However, several factors in modern society are constraining the reliance of First Nations on traditional foods. Land-use restrictions in harvesting, declines in harvests from industrial impacts and climate change, altered migration patterns of animals, competing economic priorities, and loss of knowledge of traditional hunting practices are some of the significant barriers that First Nations face (Kuhnlein and Receveur 1996; Kuhnlein et al. 2013). There is also concern about the presence of contaminants, such as metals and persistent, bioaccumulative chemicals, in animal and plant species and parts that are used as traditional foods (Seabert et al. 2014).

Due to their known adverse effects on human health, contaminants of concern include metals, such as cadmium (Cd), lead (Pb), arsenic (As), mercury (Hg), and methyl mercury (MeHg), and persistent organic pollutants, such as p,p′-dichlorodiphenyldichloroethylene (p,p′-DDE, a persistent metabolite of the insecticide DDT) and the polychlorinated biphenyls (PCBs). Lead, for example, has been shown to cause neurodevelopmental impairment in children, and mercury has been linked to neurological dysfunction and increased blood pressure (Bellinger 2008; Rice et al. 2014; Hu et al. 2018). The pesticide metabolite, p,p′-DDE, is an organochlorine that accumulates in fat tissue and has been associated with a range of adverse effects, such as cancer, diabetes, and hypertension (Emeville et al. 2015; Van Larebeke et al. 2015; Singh and Chan 2017). Studies have suggested that PCBs may affect the cardiovascular and endocrine systems (US EPA 2015).

Previous studies have examined levels of contaminants in traditional foods of First Nations in different regions of Canada south of the 60^th^ parallel (Supplementary Material A). Contaminants (e.g., mercury, PCBs) were measured in fish from the Anishinaabe tribal fisheries (Lake Huron, Lake Superior, and Lake Michigan) (Dellinger et al. 2018), from the Great Lakes region for the Ojibwa Native Americans (Chiu et al. 2004), and from Kahnawake, near Montreal (Chan et al. 1999). Also, various wild food samples of the Wapekeka and Kasabonika Lake in Northern Ontario (Seabert et al. 2014), Grassy Narrows and Whitedog First Nations in Ontario (Sellers 2010), and from Northern Quebec (Chevalier et al. 1997; Langlois et al. 1995) were assessed for metals and organochlorines. Common snapping turtle eggs in the Akwesasne, Mohawk Territory in Ontario, which were sampled 2 to 13 km downstream of a PCB-contaminated landfill site, were found to have high concentrations of 59 PCB congeners (De Solla et al. 2001). These studies were primarily conducted in localized regions on selected species and show that contaminants are present in traditional foods at varying levels.

The First Nations Food, Nutrition and Environment Study (FNFNES) provides regionally representative data from across Canada of contaminant concentrations in traditional foods of First Nation communities located south of the 60^th^ parallel. The data were collected over a 10-year period and can be used to compare contaminant concentrations and contaminant intake among First Nations in different regions of Canada. Descriptive results were presented in a draft report (Chan et al. 2019), primarily for the participating First Nations to respect the commitment to report results to First Nations first before wider release to the scientific community. The objectives of this work were to (1) rank traditional foods according to concentrations of cadmium, lead, arsenic, mercury, methyl mercury, p,p′-DDE, and total PCBs, (2) identify the key traditional foods that contributed to exposure to these contaminants, and (3) quantify daily intake levels of these contaminants and assess the risk of the exposure. These analyses were conducted for all of the participating First Nations, by eleven ecozones (Pacific Maritime, Boreal Cordillera, Montane Cordillera, Taiga Plains, Boreal Plains, Prairies, Boreal Shield, Taiga Shield, Hudson Plains, Mixedwood Plains, and Atlantic Maritime) (Chan et al. 2021) to explore regional characteristics.

## Methods

### Sample collection

Details of the design and methods of the FNFNES and the map of the ecozones are presented in Chan et al. (2021) in this special issue. The FNFNES is a participatory study developed to provide reliable information on the diet of First Nations and chemical exposure through the consumption of locally harvested foods in the 10 Canadian provinces and eight Assembly of First Nation (AFN) regions (British Columbia, Alberta, Saskatchewan, Manitoba, Ontario, Quebec-Labrador, New Brunswick-Newfoundland, and Nova Scotia-Prince Edward Island). The FNFNES was conducted in full partnership with the Assembly of First Nations and the participating communities. The participation process is described in Chan et al. (2021). Traditional food samples were collected from participating FNFNES communities based on lists developed with community representatives that included items that were (1) commonly consumed, (2) of importance for nutritional or environmental concerns, and (3) known to accumulate higher concentrations of contaminants. Communities provided up to 30 composite food samples, with each composite comprising tissue from up to five replicates. In total, 2061 food composite samples comprised of 6343 replicates, representing 221 species, were collected for analysis. A list of the food/species analyzed with the scientific names is presented in Supplementary Material B.

### Analysis

Foods collected in the AFN British Columbia and Manitoba regions were analyzed by MAXXAM Analytics, in Burnaby, BC, while foods collected in the other AFN regions were analyzed by ALS Global in Burlington, ON. Total arsenic, cadmium, and lead content were analyzed from homogenized composite samples (1 g) digested in an open vessel using a combination of nitric acid and hydrogen peroxide based on EPA 200.3/6020A (US EPA 2007). Inductively coupled plasma mass spectrometry (ICP/MS) was employed to quantify metal concentrations. The limit of detection (LOD) was at least 0.022 μg/g wet weight. Total mercury concentration was measured using US EPA Method 245.7 (US EPA 2005). Briefly, tissue samples were homogenized and subsampled prior to hot block digestion with nitric and hydrochloric acids, in combination with the addition of hydrogen peroxide followed by analysis by atomic fluorescence spectrophotometry or atomic absorption spectrophotometry. The LOD for total mercury was at least 0.002 μg/g wet weight.

A subset of the samples (*N*=656 from 98 species/parts) was also measured for methyl mercury using instrumental conditions adopted from US EPA Method 1630 (US EPA 1998). Briefly, tissue samples were digested with methanol and potassium hydroxide. A portion of the digest was analyzed by aqueous phase ethylation and purge and trap, followed by capillary gas chromatography (GC). Highly selective and sensitive detection was achieved by atomic fluorescence spectrometry (AFS) after the pyrolytic decomposition of the GC eluent. Recovery of certified reference material ranged between 70% and 130%. The LOD for methyl mercury was 0.004 μg/g wet weight.

For organochlorine analysis, another portion of the sample (6 g) was homogenized in dichloromethane (DCM) and filtered through anhydrous sodium sulphate. The extract was evaporated to 6 mL, and 5 mL was injected onto the gel permeation chromatography (GPC) column, where a fraction of the eluent was collected, concentrated, and solvent exchanged to acetone:hexane (1:1). Further clean-up was performed by eluting this extract through PSA (pressure swing adsorption) columns. The final extract was concentrated and solvent exchanged to isooctane. The analysis was performed for the p,p′-DDE and PCBs using GC-MS in selective ion monitoring (SIM) mode with an EI source. A total of 36 PCB congeners (congeners 28, 33, 37, 40, 41, 44, 49, 60, 66, 74, 87, 90, 99, 105, 118, 128, 129, 136, 137, 138, 141, 153, 156, 157, 170, 180, 183, 185, 189, 191, 193, 194, 201, 203, 206, and 209) were measured and the sum was reported as total PCB. Spiked standards and blank samples were measured for QA/QC. The LOD for DDE was 1 ng/g wet weight and at least 1 ng/g wet weight for each PCB congener.

The mean concentrations of cadmium, lead, arsenic, mercury, methyl mercury, p,p′-DDE, and PCBs in traditional food items were calculated for all ecozones combined and stratified by ecozone. The metals and methyl mercury are presented in units of micrograms per gram of fresh weight, and p,p′-DDE and PCBs in units of nanograms per gram of fresh weight. For each contaminant, the top 10 traditional food items with the highest contaminant concentrations are shown in tabular format for all ecozone analyses in Supplementary Material C, and the top 5 contributors stratified by ecozone are provided in Supplementary Material D.

### Risk assessment

The risk assessment approach followed the guidance for predicting non-cancer human health risks associated with contaminants from traditional foods (Health Canada 2018). A total of 6487 participants aged 19 years and older from 93 First Nations participated in the FNFNES for an overall participation rate of 78%. Consumption of traditional food (grams/day) was estimated by totalling the number of days in the past four seasons when consumption of a particular food item was reported, then multiplied by the age- and gender-specific portion size of the corresponding food (estimated from 24-hour recalls) and divided by 360 days (four seasons of 90 days each). Survey sample weights were used to calculate the mean *intake* of traditional foods, and bootstrap weights were used to estimate the associated 95% confidence intervals. The contribution of traditional foods to the intake of cadmium, lead, arsenic, total mercury, methyl mercury, p,p′-DDE, and PCBs was calculated by multiplying the mean contaminant concentration in a particular food item with the population-weighted mean grams of intake per day of that food item. Lower and upper bounds were calculated by multiplying the mean contaminant concentration with the lower and upper 95% confidence interval of mean grams of intake. The metals and methyl mercury are presented in units of micrograms per day, and p,p′-DDE and PCBs in units of nanograms per day.

A Food Frequency Questionnaire was used to identify traditional foods that were consumed by participants. Among consumers, daily intake of cadmium, lead, arsenic, total mercury, methyl mercury, p,p′-DDE, and PCBs through traditional foods was calculated for individual participants. For the food items consumed, the contaminant concentration in each item was multiplied by the grams of intake of that item. These values were then summed and divided by the participant’s measured (or reported, if the measurement was not available) body weight (bw) to obtain a total daily contaminant intake value by a participant. If neither measured nor reported body weight was available for a participant, this value was imputed with the mean measured body weight by gender of the sample population. A summary of the body weights of the participants is presented in Supplementary Material E. The contaminant concentration of a food item was imputed with the mean contaminant concentration of that food item from the same First Nation as the participant. If the local contaminant concentration was not available, then the contaminant concentration was imputed with the mean contaminant concentration of that food item in the same ecozone as the participant. If neither local nor ecozone concentrations were available for a food item, then the contaminant concentration was imputed with the mean *all Canada* contaminant concentration of that food item. The median, range, and 95th percentile were calculated for participant-level contaminant intake. The estimated daily contaminant intake was compared to the toxicological reference values (TRVs). The TRV is the daily dose that is deemed to be tolerable or acceptable (i.e., the dose that is “safe”), based on the assumption that a threshold dose exists at or below which toxic effects do not occur (Health Canada 2010). The TRVs were the tolerable daily intake (TDI) of 1 μg/kg/day for cadmium based on renal dysfunction, 0.5 μg/kg/day for methyl mercury (0.2 μg/kg/day for women of childbearing age) based on effects on neurological functions and development, and 0.13 μg/kg/day for the sum of non-dioxin-like PCBs based on effects on liver functions (Health Canada 2010). A total of 98 food samples were measured for both total mercury and methyl mercury. The data are presented in Supplementary Material F. Methyl mercury accounted for 100% of the total mercury in many fish and shellfish species, including halibut, rockfish, squid, ling cod, Pacific herring, sucker, octopus, lobster, shrimp, trout, scallops, salmon, ooligan, sole, and prawns. The lowest percentage of methyl mercury to total mercury was found in salmon eggs (4%). We focused the risk assessment on total mercury exposure as the dataset is more complete, but we used the TRV for methyl mercury to calculate the hazard quotient (HQ) based on a conservative assumption that 100% of the total mercury is methyl mercury in all foods. For arsenic, we used the United States Environmental Protection Agency’s Reference Dose for inorganic arsenic of 0.3 μg/kg/day based on hyperpigmentation, keratosis, and possible vascular complications (US EPA 2000). For lead, because of its lack of threshold level, we used an alternative margin of exposure (MOE) approach, which is proposed by the European Food Safety Authority (EFSA) CONTAM panel for risk characterization of dietary lead exposures (EFSA 2010). Lead exposures were compared to the level of 1.3 μg/kg/day established by the Joint FAO/WHO Expert Committee on Food Additives; this level is associated with an increase of 1 mmHg in the systolic blood pressure of adults (JECFA 2010). For DDE, we used the TDI of 10 μg/kg/day established by the FAO/WHO based on effects on development (WHO 2000). HQs were calculated by dividing the median with the TRV, and the 95th percentile with the TRV. An HQ <1 suggests that contaminant exposure poses minimal or negligible risk.

## Results

### Metals

#### Cadmium

The highest concentration of cadmium was found in the kidneys of beaver, moose, and rabbit (Table 1). When stratified by ecozone, kidney (primarily from moose) had the highest concentration of cadmium in all ecozones except the Boreal Cordillera (Supplementary Material C, Table 1). In the Boreal Cordillera, moose liver had the highest concentration of cadmium.

**Table 1:** Top 10 traditional foods with highest concentrations of metals of human health concern

Traditional food	Number of food composite	Mean (SD) (μg/g fresh weight)	Median (range) (μg/g fresh weight)
Cadmium			
1. Beaver kidney	1	21.60 (NA)	21.60 (NA)
2. Moose kidney	40	11.22 (8.85)	9.80 (0–31.10)
3. Rabbit kidney	2	6.34 (7.01)	6.34 (1.38–11.30)
4. Seaweed	5	3.99 (2.10)	4.81 (0.61–5.76)
5. Caribou kidney	4	3.89 (2.78)	4.57 (0.02–6.42)
6. Deer kidney	9	3.61 (3.13)	3.55 (0.05–8.83)
7. Moose liver	49	2.17 (1.94)	1.75 (0.01–8.46)
8. Mussels	6	2.03 (3.19)	0.56 (0.04–8.20)
9. Beaver liver	2	1.89 (2.20)	1.89 (0.33–3.44)
10. Oysters	4	1.85 (1.17)	1.45 (0.95–3.56)
Lead			
1. Bison meat	5	26.25 (58.56)	0.01 (ND–131.00)
2. Squirrel meat	5	18.57 (39.54)	1.46 (0.02–89.30)
3. Grouse meat	82	4.99 (18.77)	0.09 (ND–152.00)
4. Duck heart	2	4.67 (6.60)	4.67 (ND–9.34)
5. Rabbit/hare meat	58	4.10 (22.15)	0.01 (ND–163.00)
6. Dandelion roots	1	3.79 (NA)	3.79 (NA)
7. Beaver heart	1	2.69 (NA)	2.69 (NA)
8. Duck meat	73	1.92 (12.20)	0.03 (ND–104.00)
9. Deer meat	65	1.90 (6.77)	0.01 (ND–42.40)
10. Beaver meat	29	1.88 (9.19)	0.01 (ND–49.49)
Arsenic			
1. Seaweed	5	25.27 (13.37)	31.00 (3.45–35.10)
2. Crabs	14	9.56 (6.54)	7.83 (3.48–25.90)
3. Octopus	1	9.07 (NA)	9.07 (NA)
4. Prawns	3	8.91 (1.13)	8.48 (8.06–10.20)
5. Shad	1	7.44 (NA)	7.44 (NA)
6. Sole	2	5.78 (6.11)	5.78 (1.46–10.10)
7. Lobster	12	5.75 (3.47)	4.68 (1.61–13.80)
8. Sea cucumber	1	5.13 (NA)	5.13 (NA)
9. Flounder	2	3.74 (0.22)	3.74 (3.58–3.89)
10. Shrimp	2	3.60 (0.60)	3.60 (3.17–4.02)
Mercury			
1. Harp seal meat	1	1.06 (NA)	1.06 (NA)
2. Arctic char	1	0.92 (NA)	0.92 (NA)
3. Caribou kidney	4	0.59 (0.40)	0.72 (0.01–0.91)
4. Carp	2	0.54 (0.25)	0.54 (0.37–0.72)
5. Northern pike/jackfish	37	0.44 (0.47)	0.29 (0.04–2.75)
6. Bass	11	0.40 (0.30)	0.33 (0.11–1.07)
7. Walleye/pickerel	49	0.38 (0.25)	0.34 (0.01–1.27)
8. Sturgeon	13	0.24 (0.19)	0.19 (0.04–0.63)
9. Mushrooms	15	0.22 (0.46)	0.02 (ND–1.72)
10. Ling cod	6	0.21 (0.13)	0.18 (0.09–0.43)
Methyl mercury			
1. Harp seal meat	1	1.39 (NA)	1.39 (NA)
2. Arctic char	1	0.74 (NA)	0.74 (NA)
3. Bass	9	0.33 (0.46)	0.15 (0.05–1.53)
4. Walleye/pickerel	41	0.30 (0.31)	0.17 (0.03–1.49)
5. Northern pike/jackfish	34	0.27 (0.20)	0.21 (0.04–0.72)
6. Rockfish	6	0.24 (0.13)	0.19 (0.11–0.41)
7. Ling cod/mariah/burbot	4	0.24 (0.15)	0.25 (0.09–0.36)
8. Halibut	8	0.21 (0.11)	0.19 (0.02–0.38)
9. Trout	74	0.19 (0.20)	0.11 (0.01–0.95)
10. Sturgeon	10	0.18 (0.15)	0.15 (0.02–0.54)

*NA*, not applicable; *SD*, standard deviation

Note: All original values that were less than the detection limit were changed to zero for the contaminant analyses

Moose kidney was the most significant contributor to cadmium intake among traditional food consumers (Table 2). When stratified by ecozone, moose kidney was the main contributor to cadmium intake in the Montane Cordillera, Taiga Plains, Boreal Plains, Prairies, Boreal Shield, and Hudson Plains (Supplementary Material D, Table 1). Caribou kidney was the main contributor in the Taiga Shield and moose liver in the Boreal Cordillera. In the Atlantic Maritime, seafood (lobster, oyster, mussel, and scallop) contributed most to cadmium intake, and the contribution of moose kidney ranked fifth. In the Pacific Maritime, oyster was the highest contributor to cadmium intake, followed by seaweed.

**Table 2:** Top 10 contributors to metal intake in consumers of traditional foods

Traditional food	Percentage of total TF consumed	Bootstrap weighted Mean TF intake (95% CI) (g/day)^1^	Mean contaminant concentration in TF (μg/g)	Mean contaminant intake (95% CI) (μg/day)^2^
Cadmium				
1. Moose kidney	0.95%	0.43 (0.23–0.63)	11.22	4.86 (2.61–7.12)
2. Moose liver	1.10%	0.57 (0.32–0.82)	2.17	1.23 (0.69–1.77)
3. Mussels	0.34%	0.58 (0.08–1.08)	2.03	1.17 (0.16–2.18)
4. Seaweed	0.04%	0.19 (0–0.39)	3.99	0.77 (ND–1.56)
5. Oysters	0.19%	0.33 (0–0.69)	1.85	0.61 (ND–1.27)
6. Deer kidney	0.22%	0.13 (0.06–0.21)	3.61	0.48 (0.21–0.76)
7. Caribou kidney	0.35%	0.07 (0.02–0.13)	3.89	0.29 (0.07–0.51)
8. Lobster	0.70%	0.61 (0.51–0.72)	0.32	0.20 (0.16–0.23)
9. Herring eggs	0.30%	1.75 (0.12–3.39)	0.10	0.17 (0.01–0.33)
10. Deer liver	0.51%	0.33 (0.11–0.56)	0.38	0.13 (0.04–0.21)
Lead				
1. Bison meat	0.17%	0.27 (0.09–0.44)	26.25	6.98 (2.42–11.54)
2. Deer meat	5.82%	3.62 (1.92–5.32)	1.90	6.87 (3.64–10.09)
3. Moose meat	21.3%	9.28 (7.13–11.43)	0.35	3.20 (2.46–3.94)
4. Grouse meat	1.14%	0.44 (0.32–0.56)	4.99	2.19 (1.57–2.80)
5. Beaver meat	0.68%	0.32 (0.15–0.48)	1.88	0.60 (0.29–0.91)
6. Goose meat	3.46%	0.85 (0.42–1.29)	0.64	0.55 (0.27–0.83)
7. Elk meat	2.00%	1.67 (0.88–2.46)	0.30	0.49 (0.26–0.73)
8. Duck meat	2.85%	0.14 (0.06–0.21)	1.92	0.26 (0.11–0.41)
9. Halibut	0.79%	1.96 (0.79–3.12)	0.10	0.20 (0.08–0.32)
10. Black bear meat	0.27%	0.16 (0.03–0.29)	1.00	0.16 (0.03–0.29)
Arsenic				
1. Prawns	0.36%	1.42 (0.32–2.53)	8.91	12.70 (2.83–22.57)
2. Halibut	0.79%	1.96 (0.79–3.12)	3.01	5.89 (2.38–9.40)
3. Seaweed	0.04%	0.19 (0–0.39)	25.27	4.90 (ND–9.89)
4. Lobster	0.70%	0.61 (0.51–0.72)	5.75	3.53 (2.91–4.16)
5. Ooligan grease	0.18%	0.96 (0.04–1.89)	3.53	3.40 (0.14–6.67)
6. Shrimp	0.52%	0.81 (0.55–1.08)	3.60	2.92 (1.97–3.87)
7. Rockfish	0.32%	1.10 (0.57–1.63)	2.19	2.41 (1.25–3.57)
8. Mussels	0.34%	0.58 (0.08–1.08)	3.25	1.88 (0.26–3.50)
9. Herring eggs	0.30%	1.75 (0.12–3.39)	1.04	1.82 (0.13–3.52)
10. Crabs	0.55%	0.19 (0.10–0.28)	9.56	1.81 (0.94–2.68)
Mercury				
1. Walleye/pickerel	5.68%	3.17 (2.13–4.21)	0.38	1.21 (0.81–1.61)
2. Northern pike/jackfish	2.68%	1.05 (0.60–1.51)	0.44	0.46 (0.26–0.66)
3. Halibut	0.79%	1.96 (0.79–3.12)	0.17	0.34 (0.14–0.54)
4. Rockfish	0.32%	1.10 (0.57–1.63)	0.17	0.18 (0.10–0.27)
5. Salmon	6.21%	2.77 (1.86–3.67)	0.04	0.12 (0.08–0.16)
6. Whitefish	3.58%	0.89 (0.45–1.33)	0.10	0.08 (0.04–0.13)
7. Lobster	0.70%	0.61 (0.51–0.72)	0.13	0.08 (0.06–0.09)
8. Salmon eggs	0.49%	1.81 (0.92–2.69)	0.04	0.07 (0.03–0.10)
9. Sturgeon	0.34%	0.19 (0.11–0.27)	0.24	0.04 (0.03–0.06)
10. Caribou kidney	0.35%	0.07 (0.02–0.13)	0.59	0.04 (0.01–0.08)
Methyl mercury				
1. Walleye/pickerel	5.68%	3.17 (2.13–4.21)	0.30	0.95 (0.64–1.26)
2. Halibut	0.79%	1.96 (0.79–3.12)	0.21	0.41 (0.16–0.65)
3. Northern pike/jackfish	2.68%	1.05 (0.60–1.51)	0.27	0.29 (0.16–0.41)
4. Rockfish	0.32%	1.10 (0.57–1.63)	0.24	0.27 (0.14–0.40)
5. Salmon	6.21%	2.77 (1.86–3.67)	0.05	0.14 (0.09–0.18)
6. Lobster	0.70%	0.61 (0.51–0.72)	0.12	0.08 (0.06–0.09)
7. Whitefish	3.58%	0.89 (0.45–1.33)	0.06	0.06 (0.03–0.08)
8. Trout	3.39%	0.22 (0.14–0.30)	0.19	0.04 (0.03–0.06)
9. Sturgeon	0.34%	0.19 (0.11–0.27)	0.18	0.03 (0.02–0.05)
10. Prawns	0.36%	1.42 (0.32–2.53)	0.02	0.03 (0.01–0.06)

*CI*, confidence interval; *TF*, traditional food

^1^If the lower 95% confidence interval estimate for bootstrap weighted mean TF intake was negative, it was replaced with zero

^2^Calculated by multiplying bootstrap weighted mean TF intake with mean contaminant concentration. Estimates may not coincide exactly due to rounding of numbers in presentation

Among adults who reported eating traditional food and are categorized as “consumer,” cadmium intake ranged from ND to 15.72 μg/kg/day (Table 3). The TRV of 1 μg/kg/day was exceeded by 118 (1.9%) participants. The HQs (both median and 95th percentile) were less than one at the all ecozone level. When stratified by ecozone, none of the HQs based on the median intake exceeded one. However, the HQ (95th percentile) was 2.85 in the Boreal Cordillera, and 2.00 in the Taiga Plains. At the all ecozone level, the HQs (both median and 95th percentile) for women of childbearing age were less than one. However, when stratified by ecozone, the HQ (95th percentile) was 1.46 in the Boreal Cordillera and 1.30 in the Taiga Plains.

**Table 3:** Estimated daily dietary metal intake (μg/kg/day) and hazard quotients for traditional foods consumers

Contaminant	*N*	Median	Range	95th percentile	*N* (%)> TRV	HQ (median/TRV)	HQ(95th percentile/TRV)
Cadmium(μg/kg/day), TRV = 1							
All ecozones	6105	0.003	ND–15.72	0.39	118 (1.9)	0.003	0.39
Pacific Maritime	483	0.018	ND–4.82	0.35	6 (1.2)	0.018	0.35
Boreal Cordillera	80	0.33	0.001–6.97	2.85	11 (13.8)	0.33	**2.85**
Montane Cordillera	312	0.007	ND–4.31	0.63	12 (3.8)	0.007	0.63
Taiga Plains	150	0.010	ND–4.68	2.00	20 (13.3)	0.010	**2.00**
Boreal Plains	1203	0.001	ND–15.72	0.42	20 (1.7)	0.001	0.42
Prairies	530	ND	ND–5.41	0.08	4 (0.8)	ND	0.08
Boreal Shield	1249	0.003	ND–12.36	0.44	31 (2.5)	0.003	0.44
Taiga Shield	269	0.07	ND–5.06	0.72	10 (3.7)	0.07	0.72
Hudson Plains	320	0.004	ND–2.22	0.37	4 (1.3)	0.004	0.37
Mixedwood Plains	605	ND	ND–0.20	0.01	0 (0)	ND	0.01
Atlantic Maritime	904	0.004	ND–0.85	0.08	0 (0)	0.004	0.08
Lead(μg/kg/day), TRV = 1.3							
All ecozones	6105	0.01	ND–37.25	0.95	225 (3.7)	0.008	0.73
Pacific Maritime	483	0.03	ND–11.85	1.04	19 (3.9)	0.023	0.80
Boreal Cordillera	80	0.02	ND–0.82	0.34	0 (0)	0.015	0.26
Montane Cordillera	312	0.008	ND–8.18	0.91	14 (4.5)	0.005	0.70
Taiga Plains	150	0.013	ND–5.08	1.07	7 (4.7)	0.01	0.82
Boreal Plains	1203	0.009	ND–17.94	1.44	64 (5.3)	0.007	**1.11**
Prairies	530	ND	ND–37.25	4.14	65 (12.3)	ND	**2.36**
Boreal Shield	1249	0.03	ND–0.98	0.25	0 (0)	0.02	0.19
Taiga Shield	269	0.010	ND–29.79	0.99	45 (3.6)	0.008	0.76
Hudson Plains	320	0.018	ND–0.93	0.22	0 (0)	0.01	0.17
Mixedwood Plains	605	0.002	ND–11.23	0.18	9 (1.5)	0.0015	0.14
Atlantic Maritime	904	0.002	ND–3.40	0.09	2 (0.2)	0.0015	0.07
Arsenic(μg/kg/day), TRV = 0.3							
All ecozones	6105	0.013	ND–12.96	1.04	832 (13.6)	0.04	**3.47**
Pacific Maritime	483	0.54	ND–12.96	4.73	327 (67.7)	**1.8**	**15.8**
Boreal Cordillera	80	0.11	ND–2.62	0.82	17 (21.3)	0.37	**2.77**
Montane Cordillera	312	0.075	ND–6.72	1.01	61 (19.6)	0.25	**3.37**
Taiga Plains	150	0.01	ND–1.37	0.24	6 (4.0)	0.03	0.80
Boreal Plains	1203	0.003	ND–3.14	0.12	25 (2.1)	0.01	0.40
Prairies	530	0.0009	ND–0.30	0.04	0 (0)	0.003	0.13
Boreal Shield	1249	0.03	ND–0.84	0.26	87 (7.0)	0.10	0.87
Taiga Shield	269	0.015	ND–3.37	0.42	9 (3.3)	0.05	**1.40**
Hudson Plains	320	0.03	ND–1.56	0.34	20 (6.3)	0.10	**1.13**
Mixedwood Plains	605	0.001	ND–0.50	0.06	5 (0.8)	0.003	0.20
Atlantic Maritime	904	0.11	ND–12.00	1.81	275 (30.4)	0.37	**6.03**
Mercury(μg/kg/day), TRV = 0.5							
All ecozones	6105	0.007	ND–1.27	0.13	41 (0.7)	0.014	0.26
Pacific Maritime	483	0.015	ND–0.74	0.10	1 (0.2)	0.03	0.20
Boreal Cordillera	80	0.01	ND–0.20	0.06	0 (0)	0.02	0.12
Montane Cordillera	312	0.004	ND–0.32	0.06	0 (0)	0.008	0.12
Taiga Plains	150	0.003	ND–0.33	0.08	0 (0)	0.006	0.16
Boreal Plains	1203	0.004	ND–0.97	0.09	4 (0.3)	0.008	0.18
Prairies	530	0.0006	ND–0.23	0.03	0 (0)	0.0012	0.06
Boreal Shield	1249	0.04	ND–0.89	0.31	7 (2.6)	0.08	0.62
Taiga Shield	269	0.02	ND–1.27	0.29	28 (2.2)	0.04	0.58
Hudson Plains	320	0.01	ND–0.68	0.21	1 (0.3)	0.02	0.42
Mixedwood Plains	605	0.002	ND–0.44	0.07	0 (0)	0.004	0.14
Atlantic Maritime	904	0.003	ND–0.23	0.03	0 (0)	0.006	0.06

Bold values denote HQ>1

*HQ*, hazard quotient; *TRV*, toxicological reference value

#### Lead

Higher concentrations of lead were detected in samples of meat from bison, squirrel, grouse, duck, and rabbit/hare heart (Table 1). At the ecozone level, the highest concentrations were found in samples of grouse meat in the Pacific Maritime, Taiga Plains, Boreal Shield, and Hudson Plains; deer meat in the Montane Cordillera and Mixedwood Plains; bison meat in the Boreal Plains; rabbit or hare meat in the Prairies; caribou heart and muskrat meat in the Taiga Shield; and squirrel meat in the Atlantic Maritime (Supplementary Material C, Table 2).

At the all ecozone level, the most significant traditional food contributors to lead intake were bison meat, deer meat, moose meat, grouse meat, and beaver meat (Table 2). In ecozone analyses, deer meat was the highest contributor in the Pacific Maritime, Montane Cordillera, Prairies, Mixedwood Plains, and Atlantic Maritime, grouse meat in the Taiga Plains, Taiga Shield, and Hudson Plains, bison meat in the Boreal Plains, moose meat in the Boreal Shield, and goose meat in the Hudson Plains (Supplementary Material D, Table 2).

Lead intake ranged from ND to 37.25 μg/kg/day (Table 3). The TRV of 1.3 μg/kg/day was exceeded by 225 (3.7%) participants. The HQs (both median and 95th percentile) were less than one. The Boreal Plains and Prairies had the largest number of exceedances (5.3% and 12.3%, respectively), and the HQs (95th percentile) in these ecozones exceeded one (1.11 and 2.36, respectively).

#### Arsenic

The highest concentrations of arsenic were found in seaweed, crab, octopus, prawn, and shad (Table 1). The highest concentrations of arsenic were found in fish samples in several ecozones (i.e., salmon in the Boreal Cordillera, halibut in the Montane Cordillera, Atlantic salmon in the Taiga Shield, cisco in the Hudson Plains, sturgeon in the Mixedwood Plains, and perch in the Atlantic Maritime) (Supplementary Material C, Table 3). Seaweed had the highest concentration of arsenic in the Pacific Maritime and lobster had the highest concentration in the Boreal Shield.

The main contributor to arsenic intake was prawn, followed by halibut, seaweed, lobster, and ooligan grease (Table 2). In ecozone analyses, prawn in the Pacific Maritime resulted in the highest arsenic intake (Supplementary Material D, Table 3). The species of fish that contributed the most to arsenic intake were: salmon (Boreal Cordillera, Montane Cordillera, and Mixedwood Plains), walleye/pickerel (Prairies), and whitefish (Taiga Shield and Hudson Plains). Lobster was the main contributor in the Atlantic Maritime, and mussels were the main contributors in the Boreal Shield.

Arsenic intake ranged from ND to 12.96 μg/kg/day (Table 3). The TRV of 0.3 μg/kg/day was exceeded by 832 (13.6%) participants. The HQs (the median) in all regions were less than one. However, the HQ (95th percentile) was 3.47. In the Pacific Maritimes, the HQ (median) was 1.8, and the HQ (95th percentile) was 15.8. The HQs (median) in all other regions were below 1. The other HQs (95th percentile) were above 1 in the Boreal Cordillera (2.77), Montane Cordillera (3.37), Taiga Shield (1.40), Hudson Plains (1.13), and Atlantic Maritime (6.03). Among women of childbearing age, the TRV was exceeded by 287 (11.1%) (Table 4). The HQs (both median and 95th percentile) were greater than 1 in the Pacific Maritime. Additionally, HQs (95th percentile) were close to or greater than 1 in the Boreal Cordillera, Montane Cordillera, Boreal Shield, and Atlantic Maritime.

**Table 4:** Estimated daily dietary mercury (μg/kg/day) and hazard quotients for traditional foods consumers who are women of childbearing age

Contaminant	*N*	Median (μg)	Range (μg)	95th percentile (μg)	*N* (percent) > TRV	HQ (median/TRV)	HQ (95th percentile/TRV)
Mercury(μg/kg/day), TRV = 0.2							
All ecozones	2,585	0.004	ND–0.82	0.10	50 (1.9)	0.02	0.50
Pacific Maritime	202	0.01	ND–0.21	0.07	2 (1.0)	0.05	0.35
Boreal Cordillera	46	0.007	ND–0.07	0.05	0 (0)	0.035	0.25
Montane Cordillera	135	0.003	ND–0.32	0.04	1 (0.7)	0.015	0.20
Taiga Plains	75	0.002	ND–0.33	0.06	1 (1.3)	0.01	0.30
Boreal Plains	560	0.002	ND–0.42	0.08	7 (1.3)	0.01	0.40
Prairies	205	0.0002	ND–0.20	0.01	1 (0.5)	0.001	0.05
Boreal Shield	500	0.02	ND–0.82	0.20	25 (5.0)	0.10	**1.00**
Taiga Shield	135	0.03	ND–0.78	0.28	9 (6.7)	0.15	**1.40**
Hudson Plains	149	0.007	ND–0.29	0.07	3 (2.0)	0.035	0.35
Mixedwood Plains	195	0.0007	ND–0.35	0.06	1 (0.5)	0.0035	0.30
Atlantic Maritime	383	0.002	ND–0.19	0.03	0 (0)	0.01	0.15

Bold values denote HQ>1

*HQ, *hazard quotient*; TRV, *toxicological reference value

#### Mercury and methyl mercury

Harp seal meat, Arctic char, caribou kidney, carp, and northern pike/jackfish had the highest concentrations of mercury (Table 1). In ecozone analyses, fish often had the highest mercury concentrations, such as trout in the Boreal Cordillera, Arctic char in the Montane Cordillera, northern pike/jackfish in the Taiga Plains and Hudson Plains, walleye/pickerel in the Boreal Plains and Prairies, and bass in the Atlantic Maritime (Supplementary Material C, Table 4). In the Pacific Maritime, similar concentrations of mercury were found in samples of mushrooms and halibut. The highest concentrations of mercury from the Taiga Shield and Boreal Shield were found in caribou kidney and harp seal meat, respectively. Harp seal meat, Arctic char, bass, walleye/pickerel, and northern pike/jackfish had the highest concentrations of methyl mercury (Table 1). In ecozone analyses, fish had the highest concentration of methyl mercury, except for harp seal meat in the Boreal Shield (Supplementary Material C, Table 5).

**Table 5:** Top 10 traditional foods with highest concentrations of organochlorines

Traditional food	Number of food composite	Mean (SD) (ng/g fresh weight)	Median (range) (ng/g fresh weight)
p,p′-DDE			
1. Harp seal meat	1	28.50 (NA)	28.50 (NA)
2. Ooligan grease	5	21.12 (6.22)	19.60 (15.00–30.30)
3. Beaver kidney	1	16.10 (NA)	16.10 (NA)
4. Beaver liver	1	13.80 (NA)	13.80 (NA)
5. Duck meat	25	10.36 (25.14)	1.57 (ND–102.00)
6. Catfish	6	9.74 (6.58)	12.75 (0.26–16.30)
7. Trout	75	9.34 (19.71)	2.00 (ND–109.00)
8. Bass	9	9.22 (17.43)	2.43 (ND–53.90)
9. Eel	8	8.98 (11.18)	4.38 (1.10–35.10)
10. Salmon eggs	11	7.88 (18.88)	2.17 (ND–64.30)
PCBs			
1. Harp seal meat	1	265.40 (NA)	265.40 (NA)
2. Carp	2	63.26 (89.46)	63.26 (ND–126.52)
3. Catfish	6	59.72 (89.89)	11.91 (2.60–231.17)
4. Sturgeon	13	54.11 (120.45)	4.62 (ND–351.95)
5. Duck meat	25	39.51 (120.33)	0.64 (ND–582.01)
6. Perch	10	20.66 (45.57)	7.17 (ND–149.38)
7. Trout	75	18.06 (53.86)	2.34 (ND–298.51)
8. Bass	8	17.86 (15.86)	18.77 (0.44–39.88)
9. Ptarmigan meat	1	14.75 (NA)	14.75 (NA)
10. Black bear fat	9	12.85 (25.43)	ND (ND–78.15)

*DDE*, dichlorodiphenyldichloroethylene; *PCBs*, polychlorinated biphenyls; *SD*, standard deviation

Note: All original values that were less than the detection limit were changed to zero for the contaminant analyses

Consumption of walleye/pickerel resulted in the highest intake of mercury, followed by northern pike/jackfish, halibut, rockfish, and salmon (Table 2). Similar findings were observed for the highest contributors to methyl mercury intake (Table 2). In most ecozones, the most significant contributors to mercury intake were fish, except in the Taiga Shield and Atlantic Maritime, where caribou kidney and lobster, respectively, were the highest contributors (Supplementary Material D, Table 4). Similarly, fish was the main contributor to methyl mercury intake in all ecozones except the Atlantic Maritime, where lobster led to the highest intake (Supplementary Material D, Table 5).

Mercury intake ranged from ND to 1.27 μg/kg/day (Table 3). For all ecozones, the TRV of 0.5 μg/kg/day was exceeded by 41 (0.7%) participants, and the HQs (both median and 95th percentile) were less than one. Among women of childbearing age, mercury intake ranged from ND to 0.82 μg/kg/day and 50 (1.9%) exceeded the TRV of 0.2 μg/kg/day (Table 4). The HQ (95th percentile) for women of childbearing age was 1.00 in the Boreal Shield and 1.40 in the Taiga Shield. All HQs (both median and 95th percentile) were less than one in other regions.

### Organochlorines

#### p,p′-DDE

The highest concentrations of p,p′-DDE were in harp seal meat, followed by ooligan grease, beaver kidney, beaver liver, and duck meat (Table 5). In ecozone analyses, ooligan grease had the highest concentration in the Pacific Maritime and Montane Cordillera, salmon in the Boreal Cordillera, goose meat in the Taiga Plains and Hudson Plains, beaver kidney in the Boreal Plains, deer liver in the Prairies, duck meat in the Taiga Shield, salmon eggs in the Boreal Shield, trout in the Mixedwood Plains, and bass in the Atlantic Maritime (Supplementary Material C, Table 6).

**Table 6:** Top 10 contributors to organochlorine intake in consumers of traditional foods

Traditional food	Percentage of total TF consumed	Bootstrap weighted Mean TF intake (95% CI) (g/day)^1^	Mean contaminant concentration in TF (ng/g)	Mean contaminant intake (95% CI) (ng/day)^2^
p,p′-DDE				
1. Ooligan grease	0.18%	0.96 (0.04–1.89)	21.12	20.36 (0.81–39.91)
2. Salmon	6.21%	2.77 (1.86–3.67)	5.57	15.42 (10.39–20.46)
3. Salmon eggs	0.49%	1.81 (0.92–2.69)	7.88	14.23 (7.27–21.20)
4. Goose meat	3.46%	0.85 (0.42–1.29)	4.86	4.15 (2.02–6.28)
5. Walleye/pickerel	5.68%	3.17 (2.13–4.21)	1.07	3.40 (2.28–4.52)
6. Halibut	0.79%	1.96 (0.79–3.12)	1.66	3.25 (1.31–5.18)
7. Whitefish	3.58%	0.89 (0.45–1.33)	2.64	2.35 (1.20–3.51)
8. Trout	3.39%	0.22 (0.14–0.30)	9.34	2.05 (1.33–2.77)
9. Atlantic salmon	0.42%	0.38 (0.31–0.46)	5.30	2.03 (1.63–2.44)
10. Ooligan	0.18%	0.79 (0.25–1.33)	2.54	2.00 (0.63–3.38)
PCBs				
1. Salmon	6.21%	2.77 (1.86–3.67)	9.27	25.66 (17.28–34.04)
2. Salmon eggs	0.49%	1.81 (0.92–2.69)	10.84	19.58 (10.00–29.17)
3. Walleye/pickerel	5.68%	3.17 (2.13–4.21)	5.47	17.34 (11.65–23.04)
4. Sturgeon	0.34%	0.19 (0.11–0.27)	54.11	10.20 (5.81–14.59)
5. Ptarmigan meat	0.29%	0.42 (0–0.88)	14.75	6.23 (ND–13.05)
6. Duck meat	2.85%	0.14 (0.06–0.21)	39.51	5.34 (2.29–8.39)
7. Whitefish	3.58%	0.89 (0.45–1.33)	5.31	4.73 (2.41–7.05)
8. Perch	0.39%	0.20 (0.10–0.30)	20.66	4.13 (2.11–6.16)
9. Trout	3.39%	0.22 (0.14–0.30)	18.06	3.96 (2.58–5.35)
10. Pacific herring	0.14%	0.33 (0.02–0.64)	8.24	2.72 (0.19–5.25)

*CI*, confidence interval; *DDE*, dichlorodiphenyldichloroethylene; *PCBs*, polychlorinated biphenyls; *TF*, traditional food

^1^If the lower 95% confidence interval estimate for bootstrap weighted mean TF intake was negative, it was replaced with zero

^2^Calculated by multiplying bootstrap weighted mean TF intake with mean contaminant concentration. Estimates may not coincide exactly due to rounding of numbers in presentation

The main contributors to p,p′-DDE intake were ooligan grease, salmon, salmon eggs, goose meat, and walleye/pickerel (Table 6). Ooligan grease was the most significant contributor in the Pacific Maritime, while salmon was the highest contributor in the Boreal Cordillera, Montane Cordillera, Mixedwood Plains, and the Atlantic Maritime (Supplementary Material D, Table 6). Walleye/pickerel was the highest contributor in the Boreal Shield, and trout in the Taiga Shield. Goose meat was the most significant contributor in the Taiga Plains and Hudson Plains, moose meat in the Boreal Plains, and deer liver in the Prairies. The intake of p,p′-DDE ranged from ND to 86.86 ng/kg/day. No participant exceeded the TRV and HQs (both median and 95th percentile) were less than one (Table 7).

**Table 7:** Estimated daily dietary organochlorine intake (ng/kg/day) and hazard quotients for traditional foods consumers

Contaminant	*N*	Median	Range	95th percentile	*N* (%) > TRV	HQ (median/TRV)	HQ (95th percentile/TRV)
p,p′-DDE (ng/kg/day), TRV = 10,000							
All ecozones	6105	0.11	ND–86.86	3.07	0 (0)	1.1 × 10^−5^	3.1 × 10^−4^
Pacific Maritime	483	0.79	ND–19.03	5.15	0 (0)	7.9 × 10^−5^	5.2 × 10^−4^
Boreal Cordillera	80	0.02	ND–1.39	0.77	0 (0)	2.0 × 10^−6^	7.7 × 10^−5^
Montane Cordillera	312	0.05	ND–10.57	2.51	0 (0)	5.0 × 10^−6^	2.5 × 10^−4^
Taiga Plains	150	0.06	ND–6.59	2.47	0 (0)	6.0 × 10^−6^	2.5 × 10^−4^
Boreal Plains	1203	0.06	ND–10.94	1.58	0 (0)	6.0 × 10^−6^	1.6 × 10^−4^
Prairies	530	ND	ND–10.43	0.80	0 (0)	0	8.0 × 10^−5^
Boreal Shield	1249	0.43	ND–13.87	3.61	0 (0)	4.3 × 10^−5^	3.6 × 10^−4^
Taiga Shield	269	0.19	ND–25.87	3.93	0 (0)	1.9 × 10^−5^	3.9 × 10^−4^
Hudson Plains	320	1.07	ND–86.86	21.05	0 (0)	1.1 × 10^−4^	2.1 × 10^−3^
Mixedwood Plains	605	0.02	ND–36.99	2.42	0 (0)	2.0 × 10^−6^	2.4 × 10^−4^
Atlantic Maritime	904	0.08	ND–6.32	1.14	0 (0)	8.0 × 10^−6^	1.1 × 10^−4^
PCBs (ng/kg/day), TRV = 130							
All ecozones	6105	0.08	ND–111.14	4.72	0 (0)	6.2 × 10^−4^	0.04
Pacific Maritime	483	0.21	ND–10.91	1.79	0 (0)	1.6 × 10^−3^	0.01
Boreal Cordillera	80	0.006	ND–0.69	0.62	0 (0)	4.6 × 10^−5^	4.7 × 10^−3^
Montane Cordillera	312	ND	ND–8.52	0.49	0 (0)	0	3.7 × 10^−3^
Taiga Plains	150	0.01	ND–9.84	1.26	0 (0)	7.7 × 10^−5^	9.7 × 10^−3^
Boreal Plains	1203	ND	ND–51.98	3.08	0 (0)	0	0.02
Prairies	530	ND	ND–9.11	0.51	0 (0)	0	3.9 × 10^−3^
Boreal Shield	1249	0.64	ND–20.47	5.90	0 (0)	4.9 × 10^−3^	0.05
Taiga Shield	269	0.47	ND–101.41	10.69	0 (0)	3.6 × 10^−3^	0.08
Hudson Plains	320	0.11	ND–7.61	1.19	0 (0)	8.5 × 10^−4^	9.2 × 10^−3^
Mixedwood Plains	605	0.09	ND–111.14	13.38	0 (0)	6.9 × 10^−4^	0.10
Atlantic Maritime	904	0.085	ND–8.88	1.55	0 (0)	6.5 × 10^−4^	0.01

*DDE*, dichlorodiphenyldichloroethylene; *HQ*, hazard quotient; *PCB*, polychlorinated biphenyl; *TRV*, toxicological reference value

#### Total PCBs

PCBs were highest in harp seal meat, carp, catfish, sturgeon, and duck meat (Table 5). In ecozone analyses, PCBs were highest in herring in the Pacific Maritime, Arctic char in the Montane Cordillera, salmon in the Taiga Plains, duck meat in the Boreal Plains and Taiga Shield, whitefish in the Prairies, harp seal meat in the Boreal Shield, black bear fat in the Hudson Plains, sturgeon in the Mixedwood Plains, and bass in the Atlantic Maritime (Supplementary Material C, Table 7). Salmon, salmon eggs, walleye/pickerel, sturgeon, and ptarmigan meat were the main contributors to PCB intake (Table 6). Fish was the main contributor to PCB intake in most ecozones (Supplementary Material D, Table 7).

PCB intake ranged from ND to 111.14 ng/kg/day. No participant exceeded the TRV, and HQs (both median and 95th percentile) were less than one (Table 7).

## Discussion

This is the most comprehensive study of contaminants in traditional food consumed by First Nations in Canada and based on the analysis of 2061 composite food samples and intake data of 6487 participants. The results are based on aggregated data from food samples and dietary information collected from 93 First Nations across Canada. Analyses were conducted based on ecozones (*N* = 11). The concentrations of contaminants were generally within the expected range previously found in traditional foods in Canada (e.g., Chan 1998; Kuhnlein and Chan 2000; Van Oostdam et al. 2005). This large dataset provides a reference for risk assessors to estimate the baseline concentrations of contaminants typically found in each type of traditional food. In the absence of the availability of site-specific data, risk assessors can use the reported concentrations and/or the daily intake rate of this study as a proxy for hazard identification and design the list of food items to be included in a local sampling program.

Cadmium intakes were generally higher in two types of traditional foods—organ meats from land mammals such as the kidneys of beaver, moose, deer, and caribou, and bivalve molluscs such as oysters and mussels. Levels of cadmium are higher in organ meats as it accumulates in the liver and kidneys, where it is primarily bound to metallothionein (ATSDR 2012). Cadmium concentrations in moose kidneys in this study were within ranges observed in the kidney cortex of moose from Alaska (Arnold 2006) and Quebec (Crete et al. 1987). Moose livers and kidneys are very commonly consumed across communities and samples were provided by close to half (40) of the communities (Table 1). The concentrations in moose kidneys (11.22 μg/g) (Table 1) were about 400 times higher compared with the concentration of cadmium in organ meats reported in the Canadian Total Diet Study (0.0284 μg/g) (Health Canada 2011). Even though moose kidney contributed less than 1% of the traditional food diet and the daily consumption rate was very low at 0.43 g/day, it was the highest contributor to cadmium intake at 4.86 μg/day (Table 2). For the bivalves, elevated cadmium concentrations are largely attributed to natural global ocean currents in the Northern Pacific ocean (Bruland 1980) or local pollution (Bendell 2010). The mean cadmium concentration for oysters used in this assessment was 1.85 μg/g, a value within previously reported concentrations in British Columbia of 0.5–4.9 μg/g (Bendell 2010) and is similar to the concentration used by the Canadian Food Inspection Agency (2.63 μg/g) in their risk assessment report (Bendell 2010). Only 1.9% of the daily intake of cadmium among the participants exceeded the TRV, suggesting that most (98%) of the traditional food consumers would have minimal risk from cadmium intake. The HQs estimated for the median exposure were all below 1, and the HQ for the 95th centile exposure was higher than 1 in Boreal Cordillera and Taiga Plains. These results suggest that frequent consumers of land mammal organs in northern BC and Alberta may need to be more cautious about cadmium intake and should limit the consumption of a meal of moose kidney to once every 1–2 months. Chronic cadmium exposures can lead to accumulation in the kidneys, potentially impairing functionality, as well as leading to brittleness and fragility of bones in humans. Since cigarettes are a major source of cadmium exposure (ATSDR 2012), moose organ consumers who also smoke cigarettes are at an even higher risk of exposure.

Exposure to lead continues to be a public health concern despite a decline in exposure levels since the 1970s. The primary driver of the current concern is the growing body of evidence suggesting that there is no threshold for adverse effects, such as neurological deficits in children and elevated blood pressure in adults (Lanphear et al. 2005). Lead levels were higher in muscle meat, with a large range (several orders of magnitude) of lead concentrations (Table 1), suggesting contamination from lead-containing ammunition rather than a reflection of environmental lead accumulation in game species. However, since the hunting method was not recorded, and lead isotope analysis was not conducted to differentiate lead sources, conclusions regarding the sources of lead warrant further study. The HQs estimated for the median exposure were all below 1, and the HQ for the 95th percentile exposure was higher than 1 in Boreal Plains and Prairies. These results suggest that risk communication efforts, along with cost-effective and acceptable safe ammunition options, are needed. We have previously estimated that most of the lead (72.7%) in the total diet of First Nations in Ontario was through traditional food sources (Juric et al. 2018). Another factor that may contribute to the body burden is contaminated drinking water. In the FNFNES analysis of metals in tap water, few samples had lead concentrations exceeding guidelines, and these were predominantly where the sample was the first draw after the water had been stagnant in the pipes overnight. Upon running water for 5 minutes to flush the pipes, lead levels were observed to be acceptable (Schwartz et al. 2021).

Chronic arsenic exposure at a dose as little as 0.05 mg/kg body weight has a systemic effect on the human body, i.e., cardiovascular, skin pulmonary, and endocrinal effects, and can lead to cancer in multiple organs (ATSDR 2007). Risk assessment for cancer is based on exposure to inorganic arsenic (Health Canada 2010). However, in this study, we only measured total arsenic in the collected food samples and did not measure arsenic species. Therefore, we used the TRV for non-cancer endpoints to assess the risk of arsenic intake. The top ten traditional foods that had the highest arsenic concentrations were all seafood items. Fish and seafood are known to accumulate arsenic from the ocean and are the major sources of arsenic in Canadian diets (Dabeka et al. 1993). Seaweed had the highest concentration and was the most significant contributor. Arsenic exists in fish and seafood, mainly as arsenobetaine, which is non-toxic in humans and is rapidly excreted in the urine after ingestion (ATSDR 2007). The percentage of inorganic arsenic in fish and seafood is usually less than 2% but can reach as high as 30% in seaweed (Mania et al. 2015). Therefore, our assessment of the risk of arsenic exposure based on the assumption that all the arsenic is in the inorganic form is likely to be too conservative. It is not surprising that the 95th centile of participants in the Pacific Maritime and the Atlantic Maritime had a HQ of 15.8 and 6.03, respectively. Further characterization of the risk among the coastal First Nations by measuring arsenic species in seafood, particularly in seaweed, is needed.

The highest levels of mercury and methyl mercury were found in marine mammals, fish, caribou kidney, and mushrooms. The average concentrations of total mercury and methyl mercury in fish samples collected through the FNFNES were below the Health Canada guideline (0.5 μg/g) for total mercury. The highest level was over 1 μg/g in one sample of harp seal meat and in samples of freshwater fish at higher trophic levels such as walleye/pickerel and northern pike. Both fish species are important traditional foods for many First Nations, accounting for 6% (walleye) and 3% (pike) of the traditional food intake; thus, they are also the top contributors of mercury intake. Since the fetus is more susceptible to mercury toxicity, the TRV for women of childbearing age is lower at 0.2 μg/kg/day compared with other adults at 0.5 μg/kg/day (Legrand et al. 2005). While the HQ for all participants in all ecozones was below 1, the 95th percentile of mercury intake among women of childbearing age in the Boreal Shield and the Taiga Plains was 1 and 1.4, respectively. These results are in agreement with the hair mercury monitoring results, which found that a higher percentage of women of childbearing age in First Nations in northern Saskatchewan, Manitoba, Ontario, and across Quebec had hair mercury concentrations above the cut-off level of 2 μg/g (Tikhonov et al. 2021), suggesting that other culturally appropriate and acceptable strategies to enable ongoing fish consumption, while reducing mercury exposure, need to be investigated.

The use of organochlorines has been banned for decades, but because of their long half-life in the environment, these legacy chemicals are still detected in fish and wildlife and remain a health concern (Health Canada 2004). Since they are lipophilic compounds, they accumulate at higher concentrations in high-fat foods and animal species at higher trophic levels in the aquatic systems. Therefore, higher concentrations of p,p′-DDE and PCBs were found in marine mammals and fish samples such as harp seal meat, ooligan grease, and salmon. However, there were no traditional food consumers who exceeded the TRVs for p,p′-DDE or PCBs and the estimated HQs were all below 1, even among the 95th percentile intake. Therefore, the risk of exposure to these chemicals may no longer be a concern. However, we have found that dietary DDE and PCB intakes were positively associated with type 2 diabetes among First Nations (Marushka et al. 2017, 2021). These legacy contaminants may still need to be taken into account for the risk-benefit assessment of local fish and wildlife, particularly in known hotspots such as the Great Lakes region.

The strengths of this paper include the large number of traditional foods collected and measured for contaminant levels, the comprehensiveness of the food use data, and the representativeness of randomly selected samples at both the regional and the ecozone levels. There are also some limitations. The findings of this study provide an overview of the risk of contaminant exposure among adult First Nations living on-reserve. However, every First Nation has unique circumstances (e.g., a local pollution source), and more local data collected more frequently are needed to characterize First Nations specific risk. Also, the data were collected over a 10-year period. We have adjusted for the temporal changes in population size only. Other potential environmental, demographic, or socio-economic changes such as the contaminant concentrations or food use frequency may have occurred but have not been addressed. Furthermore, imputations of data were performed, as is the norm in risk assessment. A total of 767,737 observations were reported in the Food Frequency Questionnaire by the participants. Community-specific food contaminant concentrations data were only available for 25% of the observations. Therefore, we had to impute with contaminant concentrations data for foods collected either from the same ecozone (32%) or with the study average (43%). This has resulted in uncertainties in the risk assessment. Finally, this study did not cover the First Nations located north of the 60^th^ parallel.

## Conclusion

Contaminant concentrations found in traditional foods were generally within the expected range previously found in similar regions in Canada, except for lead. The risks of contaminant intake from traditional foods, at the rate of consumption reported in the last 10 years during the FNFNES study period, were generally low among most First Nations. Traditional foods are preferred by First Nation communities, and most would like to have them more often and in larger quantities (Chan et al. 2019). Access to traditional foods and increased local food production should be promoted with ongoing monitoring of the health of species and their contaminant burdens. The ranking of the traditional foods according to their contribution to total contaminant exposure in each ecozone is useful to identify the source so that intervention such as targeted advisories can be issued. The results also provide guidance for prioritizing the selection of traditional food items for future monitoring programs. A monitoring program of this nature should be undertaken in close consultation with and involvement of First Nations to ensure that it is relevant and sensitive to the needs of communities. The research team has been engaging First Nations and the relevant health authorities on the knowledge translation of these results to promote nutrition and environmental health.

## Supplementary information

ESM 1 (DOCX 20 kb)

ESM 2 (DOCX 36 kb)

ESM 3 (DOCX 75 kb)

ESM 4 (DOCX 60 kb)

ESM 5 (DOCX 12 kb)

ESM 6 (DOCX 23 kb)

## Data Availability

Data are owned by each participating community. The Assembly of First Nations is data custodian and any requests will be addressed to AFN through the corresponding author.

## Code availability

Analysis codes can be shared upon reasonable request.
