# Supplementary material for: Levels of metals and persistent organic pollutants in traditional foods consumed by First Nations living on-reserve in Canada
Source: Can J Public Health. 2021 Jun 28;112(Suppl 1):81–96. doi: 10.17269/s41997-021-00495-7 (PMC8239065; doi:10.17269/s41997-021-00495-7)
Supplement: Supplementary file 1 — (DOCX 20 kb) [file 41997_2021_495_MOESM1_ESM.docx]

**Supplementary Material A: Literature Review**

**Table 1: Contaminant levels in traditional foods of First Nations in Canada**

| **Author (Year)**  **Location** | **Traditional Food/Species** | **Contaminant** | **Concentration** |
| --- | --- | --- | --- |
| Dellinger (2018)  Upper Great Lakes (Anishinaabe Great Lake Native American tribal fisheries) | Fish | PCBs  Hg | **Lake Huron (mean ± SD, ng/g for PCBs and µg/g for Hg)**  **Walleye:** PCBs: 67 ± 98; Hg: 0.436 ± 0.194  **Herring:** PCBs: 26 ± 6; Hg: 0.068 ± 0.014  **Perch:** PCBs: 3 ± 2; Hg: 0.069 ± 0.040  **Whitefish:** PCBs: 34 ± 15; Hg: 0.084 ± 0.036  **Lake trout:** PCBs: 79 ± 29; Hg: 0.118 ± 0.026  **Lake Superior (mean ± SD, ng/g for PCBs and µg/g for Hg)**  **Smelt:** PCBs: 11 ± 1; Hg: 0.065 ± 0.009  **Whitefish:** PCBs: 6 ± 2; Hg: 0.059 ± 0.014  **Lake trout:** PCBs: 40 ± 16; Hg: 0.122 ± 0.041  **Lake Michigan (mean ± SD, ng/g for PCBs and µg/g for Hg)**  **Whitefish:** PCBs: 31 ± 13; Hg: 0.127 ± 0.031  **Lake trout:** PCBs: 138 ± 64; Hg: 0.133 ± 0.042 |
| Seabert (2014)  Northern Ontario (Wapekeka and Kasabonika Lake First Nations) | Wild food samples (e.g., fish, birds, beaver, rabbit) | PCBs  Hg | **Fish Muscle (ng/g for PCBs and µg/g for Hg):**  **walleye, pike, sucker, whitefish, trout:** PCBs <12;  **walleye, pike, trout:** Hg >0.120**; sucker:** Hg >0.078**; whitefish:** Hg <0.059  **Fish Organ (ng/g for PCBs and µg/g for Hg):**  **walleye:** PCBs >12; **pike, sucker, whitefish, trout:** PCBs <12;  **walleye, trout:** Hg >0.078; **pike, sucker:** Hg <0.059; **whitefish:** Hg >0.059  **Bird Muscle (ng/g for PCBs and µg/g for Hg):**  **goose, pintail:** PCBs <12; **mallard:** PCBs ≈ 40; **bluebill:** PCBs >12;  **goose, mallard, pintail:** Hg <0.059; **bluebill:** Hg >0.078  **Bird Organ (ng/g for PCBs and µg/g for Hg):**  **goose, pintail, mallard, bluebill:** PCBs < 12; **goose, pintail:** Hg <0.059; **mallard:** Hg >0.078; **bluebill:** Hg >0.120  **Beaver (ng/g for PCBs and µg/g for Hg):** organ PCBs <12; organ and muscle Hg <0.059  **Rabbit (ng/g):** organ PCBs > 80 |
| Sellers (2010)  Grassy Narrows and Whitedog First Nations, Ontario, Canada | Fish  Deer  Moose  Mink  Otter  Beaver  Marten  Rabbit  Ruffed grouse | Heavy metals:   - Cd - Hg | **Fish (range, µg/g)**  **Walleye -pickerel:** Hg: 0.32 - 0.89 (flesh)  **Northern pike:** Hg: 0.26 - 0.65 (flesh)  **Deer (range, µg/g)**  Flesh: Cd: <0.01 - 0.01; Hg: <0.005 - 0.072;  Kidney: Cd: 2.4-18; Hg: 0.18 - 0.86;  Liver: Hg: <0.005 - 0.018  **Moose (range, µg/g)**  Flesh: Cd: <0.01 - 0.01; Hg: <0.005 - 0.008;  Kidney: Cd: 2.1 - 15; Hg: 0.017 - 0.11;  Liver: Cd: 0.35; Hg: <0.005 - 0.025  **Mink (range, µg/g)** Hg: 0.49 - 4.4 (flesh)  **Otter (range, µg/g)**  Flesh: Hg: 0.35 - 1.7;  Kidney: Hg: 1.7;  Liver: Hg: 2.3 - 2.4  **Beaver (range, µg/g)** Hg: <0.005 (flesh)  **Marten (range, µg/g)** Hg: <0.005 - 0.38 (flesh)  **Rabbit (range, µg/g)** Hg: <0.005-0.016 (flesh)  **Ruffed grouse (range, µg/g)** Hg: <0.005 (flesh) |
| Chiu (2004)  Great Lakes (Ojibwa Native Americans) | Fish | PCBs | **Lake trout** **(mean ∑PCB, ng/g):** 3.42 **Walleye (mean ∑PCB, ng/g):** 3.14 **Whitefish (mean ∑PCB, ng/g):** 2.85  **Other fish (mean ∑PCB, ng/g):** 3.08 |
| De Solla (2001)  Ontario (Akwesasne, Mohawk Territory) | Common snapping turtle eggs | OCPs  PCBs  Dibenzodioxins  Furans | Total PCBs (59 congeners, range, ng/g):  2 378.2 - 737 683  Non-ortho PCBs (6 congeners, ng/g): 54.54  Dioxin + Furan (11 congeners, ng/g): 85.8  OCPs (range, ng/g): 28 – 2 264 |
| Chan (1999)  Kahnawake (near Montreal) | Fish | Organochlorines   - PCBs - Chlorobenzene - Lindane - Dieldrin - Chlordane - Heptachlor epoxide - DDT - Mirex   Heavy metals   - As - Cd - Pb - Hg | **Bullhead (mean ± SD, ng/g for organochlorines and µg/g for metals)**  PCB: 29.23 ± 14.09; Chlorobenzene: 0.07 ± 0.15; Lindane: 2.22 ± 1.89; Dieldrin: 1.86 ± 0.96; Chlordane: 4.50 ± 3.37; Heptachlor epoxide: 0.16 ± 0.20; DDT: 23.15 ± 16.62; Mirex: 0.27 ± 0.19; As: 0.0088 ± 0.0061;  Cd: 0.0014 ± 0.0010; Pb: 0.0177 ± 0.0043; Hg: 0.26 ± 0.14  **Perch (mean ± SD, ng/g for organochlorines and µg/g for metals)**  PCB: 65.94 ± 61.54; Chlorobenzene: 0.05 ± 0.15; Lindane: 4.25 ± 2.35; Dieldrin: 2.35 ± 0.96; Chlordane: 4.34 ± 2.42; Heptachlor epoxide: 0.24 ± 0.32; DDT: 20.69 ± 9.41; Mirex: 0.57 ± 0.61; As: 0.0118 ± 0.0097; Cd: 0.0012 ± 0.0007; Pb: 0.0313 ± 0.0068; Hg: 0.30 ± 0.12  **Pike (mean ± SD, ng/g for organochlorines and µg/g for metals)**  PCB: 49.92 ± 52.61; Chlorobenzene: 0.12 ± 0.21; Lindane: 3.79 ± 3.62; Dieldrin: 1.69 ± 0.50; Chlordane: 6.05 ± 4.85; Heptachlor epoxide: 0.15 ± 0.18; DDT: 29.58 ± 23.57; Mirex: 0.47 ± 0.36; As: 0.0262 ± 0.0156; Cd: 0.0008 ± 0.0010; Pb: 0.0229 ± 0.0083; Hg: 0.46 ± 0.31  **Smallmouth bass (mean ± SD, µg/g)**  As: 0.0777 ± 0.0262; Cd: 0.0089 ± 0.0181; Pb: 0.0164 ± 0.0061; Hg: 0.53 ± 0.23  **Sturgeon (mean ± SD, µg/g)**  As: 0.3702 ± 0.1217; Cd: 0.0011 ± 0.0014; Pb: 0.0126 ± 0.0052; Hg: 0.14 ± 0.07  **Walleye (mean ± SD, µg/g)**  As: 0.0579 ± 0.0166; Cd: 0.0014 ± 0.0031; Pb: 0.0144 ± 0.0062; Hg: 0.42 ± 0.38 |
| Chevalier (1997) and Langlois (1995)  Northern Quebec (Great Whale area 55^o^N, and Nottaway-Broadback-Rupert area between 49^o^N and 52^o^N) | Fish  Bird  Mink | Hg | **mean ± SD (µg/g)**  **Northern pike:** 0.93 ± 0.51 (muscle)  **Walleye:** 0.78 ± 0.46 (muscle)  **Lake trout:** 0.77 ± 0.66 (muscle)  **Common merganser:** 1.41 ± 1.26 (muscle); 17.53 ± 12.06 (liver); 9.97 ± 2.88 (feathers)  **Herring gull:** 1.59 ± 1.32 (muscle); 3.63 ± 2.52 (liver);  19.1 ± 15.3 (feathers)  **American mink:** 2.40 ± 2.24 (muscle) |

As = arsenic; Cd = cadmium; DDT = dichlorodiphenyltrichloroethane; Hg = mercury; N/A = not available; NWT = Northwest Territories; OCP = organochlorine pesticide; Pb = lead; PCB = polychlorinated biphenyl

* Other elements that were measured in the study, but not presented here: Ca, Fe, Mn, Co, Cu, Zn, Br, Rb, and Sr.
