# Supplementary material for: Levels of metals and persistent organic pollutants in traditional foods consumed by First Nations living on-reserve in Canada
Source: Can J Public Health. 2021 Jun 28;112(Suppl 1):81–96. doi: 10.17269/s41997-021-00495-7 (PMC8239065; doi:10.17269/s41997-021-00495-7)
Supplement: Supplementary file 2 — (DOCX 36 kb) [file 41997_2021_495_MOESM2_ESM.docx]

**Supplementary Material B**

**List of FNFNES traditional food samples collected for contaminant analyses**

Notes: The species list presented in this appendix was developed by cross-checking the name of the food item documented on the sample list against the field guides and the best information on hand. However, at the time of collection, there was no process to verify with the provider of the sample, which species was actually provided.

A total of 2061 food samples (comprised of 6343 replicates) from 221 species were collected for contaminant analyses.

| **Species** | **Scientific name** |
| --- | --- |
| **FISH, SEAFOOD AND MARINE MAMMALS** | |
| Abalone | *Haliotis kamtschatkana* |
| American eel | *Anguilla rostrata* |
| Arctic grayling | *Thymallus arcticus arcticus* |
| Bass, largemouth | *Micropterus dolomieu* |
| Bass, smallmouth | *Micropterus salmoides* |
| Bass, striped | *Morone saxatilis* |
| Bass, white | *Morone spp.* |
| Carp | *Cyprinus carpio* |
| Catfish | *Ameiurus nebulosus* |
| Cattail | *Typha latifolia* |
| Cisco | *Coregonus spp.* |
| Clam, butter | *Saxidomus giganteus* |
| Clam, manila | *Venerupis philippinarum* |
| Clam, razor | *Ensis directus* |
| Clam, softshell | *Mya arenaria* |
| Clams, quahog (surf clam) | *Spisula solidissima* |
| Cockles | *Clinocardium nuttalli* |
| Cod, Atlantic | *Gadus morhua* |
| Cod, black | *Anoplopoma fimbria* |
| Cod, tom | *Microgadus tomcod* |
| Crab, dungeness | *Cancer magister* |
| Crab, snow | *Chionoecetes opilio* |
| Eulachon/ooligan | *Thaleichthys pacificus* |
| Flounder | *Platichtys stellatus* |
| Gaspereau | *Alosa pseudoharengus* |
| Haddock | *Melanogrammus aeglefinus* |
| Halibut | *Hippoglussus stenolepsis* |
| Herring, Atlantic | *Clupea harengus* |
| Herring, Pacific | *Clupea pallasi* |
| Ling cod/mariah/burbot | *Lota lota* |
| Lobster | *Homarus americanus* |
| Mackerel | *Scomber scombrus* |
| Mooneye/goldeye | *Hiodon tergisus, Hiodon alosoides* |
| Mussels | *Mytilus* *spp.* |
| Northern pike/jackfish | *Esox lucius* |
| Octopus | *Octopus spp.* |
| Oysters | *Giganteus pacificus* |
| Perch, yellow | *Perca flavescens* |
| Rockfish | *Sebastes spp.* |
| Salmon, Atlantic | *Salmo salar* |
| Salmon, chinook/spring/king | *Oncorhynchus tshawytscha* |
| Salmon, chum | *Oncorhynchus keta* |
| Salmon, coho | *Oncorhynchus kisutch* |
| Salmon, pink | *Oncorhynchus gorbuscha* |
| Salmon, sockeye | *Oncorhynchus nerka* |
| Sauger | *Stizostedion canadense* |
| Scallop, Atlantic | *Pecten magellanicus* |
| Scallop, rock | *Crassadoma gigantea* |
| Sea cucumber | *Parastichopus californicus* |
| Sea snail | unidentified |
| Seal, harp | *Pagophilus groenlandicus* |
| Seaweed | *Porphyra abbottiae* |
| Shad | *Alosa sapidissima* |
| Shrimp/Prawn | *Aeginella longicornis , Bentheogennema borealis, Caprella laeviuscula, Pandulus spp.* |
| Smelt | *Osmerus mordax* |
| Sole | *Parophrys vetulus* |
| Squid | *Illex illecebrosus* |
| Sturgeon | *Acipenserspp.* |
| Sucker, longnose | *Catostomus catostomus* |
| Sucker, white | *Catostomus commersonii* |
| Trout, brook/speckled | *Salvelinus fontinalis* |
| Trout, brown | *Salmo trutta* |
| Trout, cutthroat | *Salmo clarki clarki* |
| Trout, dolly varden | *Salvelinus malma* |
| Trout, lake | *Salvelinus namaycush* |
| Trout, splake | *Salvelinus fontinalis × Salvelinus namaycush* |
| Char, arctic | *Salvelinus alpinus* |
| Trout, kokanee | *Oncorhynchus nerka* |
| Trout, rainbow/steelhead | *Oncorhynchus mykiss* |
| Trout, steelhead | *Oncorhynchusmykiss* |
| Walleye/pickerel | *Sanders vitreus* |
| Whitefish, lake | *Coregonus clupeaformis* |
| Whitefish, round | *Prosopium cylindraceum* |
| **LAND MAMMALS** | |
| Beaver | *Castor canadensis* |
| Bison | *Bison bison athabascae* |
| Black bear | *Ursus americanus* |
| Caribou | *Rangifer ssp.* |
| Deer | *Odocoileus spp.* |
| Elk | *Cervus canadensis* |
| Groundhog | *Marmota monax* |
| Hare/rabbit | *Lepus spp.* |
| Marten | *Martes americana* |
| Moose | *Alces alces* |
| Muskrat | *Ondatra zibethica* |
| Porcupine | *Erethizon dorsatum* |
| Squirrel | *Tamiasciurus hudsonicus, Sciurus carolinensis* |
| **WILD BIRDS** | |
| Arctic tern | *Sterna paradisaea* |
| Duck, American black | *Anas rubripes* |
| Duck, gadwall | *Anas strepera* |
| Duck, mallard | *Anas platyrhynchos* |
| Duck, northern shoveller | *Anas clypeata* |
| Duck, pintail | *Anas acuta* |
| Duck, teal | *Anas spp.* |
| Duck, wigeon | *Anas americana* |
| Duck, black guillemot | *Cepphus grylle* |
| Duck, bufflehead | *Bucephala albeola* |
| Duck, coot | *Fulica americana* |
| Duck, eider | *Somateria spp.* |
| Duck, godwit | *Limosa spp.* |
| Duck, goldeneye | *Bucephala clangula* |
| Duck, scaup | *Aythya marila* |
| Duck, scoter | *Melanitta nigra* |
| Duck, wood | *Aix sponsa* |
| Goose, Canada | *Branta canadensis* |
| Goose, snow | *Chen caerulescens* |
| Grouse, various spp. | *Falcipennis canadensis, Bonasa umbellus, Perdix perdix, Lagopus spp.* |
| Wild turkey | *Meleagris gallopava* |
| **BERRIES, FRUITS, NUTS AND SEEDS** | |
| Blackberry | *Rubus spp.* |
| Cloudberry/bakeapple | *Rubus chamaemorus* |
| Raspberry | *Rubus idaeus* |
| Salmonberry | *Rubus spectabilis* |
| Thimbleberry | *Rubus parviflorus* |
| Blueberry | *Vaccinium myrtilloides, Vaccinium angustifolium* |
| Bunchberry | *Cornus canadensis L.* |
| Chokecherry/pincherry | *Prunus virginiana L.* |
| Plum | *Prunus spp.* |
| Crabapple | *Malus coronaria, Pyrus coronaria* |
| Cranberry, high bush | *Viburnum spp.* |
| Cranberry, low-bush | *Vaccinium oxycoccos, Oxycoccus oxycoccos* |
| Elderberry | *Sambucus spp.* |
| Currant | *Ribes spp.* |
| Gooseberry | *Ribes spp.* |
| Hawthorn berry | *Crataegus spp.* |
| Huckleberry | *Vaccinium spp., Gaylussacia spp.* |
| Grape, Oregon | *Mahonia aquifolium* |
| Grape, wild | *Vitis riparia* |
| Rosehip | *Rosa spp.* |
| Salal berry | *Gaultheria shallon* |
| Saskatoon berry | *Amelanchier alnifolia* |
| Soapberry | *Shepherdia canadensis* |
| Strawberry | *Fragaria spp.* |
| Sumac | *Rhus typhina, R. glabra* |
| Nut, acorn | *Quercus spp.* |
| Nut, butternut | *Juglans cinerea* |
| Nut, chestnut | *Castanea dentata* |
| Nut, hazelnut | *Corylus americana* |
| Nut, hickory | *Carya ovata* |
| Nut, walnut | *Juglans spp* |
| Seeds, sunflower | *Helianthus annuus* |
| **WILD PLANTS** | |
| Asparagus | *Asparagus officinalis* |
| Avalanche lily | *Erythronium montanum* |
| Bear root | *Ligusticum spp.* |
| Bergamot, beebalm, horsemint | *Monarda spp.* |
| Scarlet beebalm (oswego) | *Monarda didyma* |
| Bitter root | *Lewisia rediviva* |
| Buck brush | *Ceanothus cuneatus* |
| Burdock | *Arctium spp.* |
| Caribou weeds | *Artemisia tilesii* |
| Clover | *Trifolium spp.* |
| Cow parsnip (Indian celery) | *Heracleum lanatum* |
| Indian celery (Indian consumption plant, desert parsley) | *Lomatium spp.* |
| Dandelion | *Taraxacum officinale* |
| Devil's club | *Oplopanax horridus* |
| Ferns, fiddleheads | *Matteauccia struthiopteris* |
| Ferns, licorice | *Polypodium glycyrrhiza* |
| Goldthread | *Coptis trifolia* |
| Horsetail shoots | *Equisetum spp.* |
| Jerusalem artichoke | *Helianthus tubererosus* |
| Labrador tea | *Ledum groenlandicum, Rhododendron groenlandicum, R.tomentosum, R. neoglandulosum* |
| Lamb's quarters | *Chenopodium album* |
| Leeks/onions | *Allium spp.* |
| Lichen-moss | *Usnea spp.* |
| Lilypad | *Nuphar lutea, Nymphaea odorata* |
| Mint | *Mentha spp.* |
| Purple pitcher (turtle socks) | *Sarracenia purpurea* |
| Sage | *Salvia spp.* |
| Stinging nettle | *Urtica dioica* |
| Strawberry blite | *Blitum capitatum* |
| Sweetflag/muskrat root | *Acorus americanus, A. calamus* |
| Sweetgrass | *Hierochloe odorata* |
| Tobacco | *Nicotiana tabacum* |
| Western dock | *Rumex occidentalis* |
| Wild ginger | *Asarum caudatum* |
| Wild parsnip | *Pastinaca sativa* |
| Wild rhubarb | unidentified |
| Wild rice | *Zitania aquatica* |
| Wintergreen (teaberry) | *Gaultheria procumbens* |
| Yarrow | *Achillea millefolium* |
| **TREES (bark, leaves, syrup, needles, cones, gum)** | |
| Alder | *Alnus incana, A. spp* |
| Balsam fir | *Abies balsamea* |
| Birch | *Betula spp.* |
| Cascara | *Rhamnus purshiana* |
| Cedar | *Thuja occidentalis, Thuja plicata* |
| Hemlock | *Tsuga canadensis* |
| Ironwood (hornbeam) | *Ostrya spp.* |
| Juniper | *Juniperus communis* |
| Maple | *Acer spp.* |
| Mountain ash | *Sorbus subg. Sorbus* |
| Pine | *Pinus strobus* |
| Poplar (balsam) | *Populus balsamifera L.* |
| Spruce | *Picea spp.* |
| Tamarack | *Larix laricina* |
| Willow | *Salix spp.* |
| Yew | *Taxus canadensis* |
| **MUSHROOMS** | |
| Mushroom, mycena | *Mycena spp.* |
| Mushroom, chaga | *Inonotus obliquus* |
| Mushroom, chanterelle | *Cantharellus spp.* |
| Mushroom, honey | *Armillaria mellea* |
| Mushroom, morel | *Morchella spp.* |
| Mushroom, pine | *Tricholoma magnivelare* |
| Mushroom, giant puffball | *Calvatia gigantea* |
| **CULTIVATED FOOD - PLANTS** | |
| Apples | *Malus domestica* |
| Beans | *Phaseolus vulgaris* |
| Beets | *Beta vulgaris* |
| Brussel sprouts | *Brassica oleracea var. gemmifera* |
| Cabbage | *Brassica oleracea var. capitata* |
| Carrots | *Daucus carota subsp. sativus* |
| Corn | *Zea mays* |
| Cucumber | *Cucumis sativus* |
| Honey | *Apis mellifera (bee)* |
| Pepper, green | *Capsicum annuum* |
| Potatoes | *Solanum tuberosum* |
| Radish | *Raphanus sativus* |
| Spinach | *Spinacia spp.* |
| Squash, summer | *Cucurbita pepo* |
| Squash, winter | *Cucurbita maxima* |
| Tomatoes | *Solanum lycopersicum* |
| Turnip | *Brassica rapa subsp. rapa* |
| **CULTIVATED FOOD - ANIMAL** | |
| Beef | *Bos taurus* |
| Eggs, chicken | *Gallus gallus domesticus* |
| Goat | *Capra aegagrus hircus* |
