# Supplementary material for: Levels of metals and persistent organic pollutants in traditional foods consumed by First Nations living on-reserve in Canada
Source: Can J Public Health. 2021 Jun 28;112(Suppl 1):81–96. doi: 10.17269/s41997-021-00495-7 (PMC8239065; doi:10.17269/s41997-021-00495-7)
Supplement: Supplementary file 3 — (DOCX 75 kb) [file 41997_2021_495_MOESM3_ESM.docx]

**Supplementary Material C**

**Traditional Food Contaminant Levels by Ecozone (N Sites = number of food composite)**

**Table 1: Cadmium**

| **Sample** | **N**  **Sites** | **Mean (ug/g)** | **SD (ug/g)** | **Median (ug/g)** | **Minimum (ug/g)** | **Maximum (ug/g)** |
| --- | --- | --- | --- | --- | --- | --- |
| **Pacific Maritime** | | | | | | |
| Moose kidney | 1 | 5.37 | - | 5.37 | 5.37 | 5.37 |
| Seaweed | 5 | 3.99 | 2.10 | 4.81 | 0.61 | 5.76 |
| Mussels | 3 | 3.67 | 4.15 | 2.75 | 0.05 | 8.20 |
| Oysters | 1 | 3.56 | - | 3.56 | 3.56 | 3.56 |
| Moose liver | 2 | 2.86 | 1.08 | 2.86 | 2.09 | 3.62 |
| **Boreal Cordillera** | | | | | | |
| Moose liver | 1 | 8.46 | - | 8.46 | 8.46 | 8.46 |
| Caribou weeds | 1 | 1.54 | - | 1.54 | 1.54 | 1.54 |
| Moose meat | 2 | 0.02 | 0.01 | 0.02 | 0.02 | 0.03 |
| Salmon | 2 | 0.01 | 0.00 | 0.01 | 0.01 | 0.01 |
| Blueberries | 1 | 0.00 | - | 0.00 | 0.00 | 0.00 |
| **Montane Cordillera** | | | | | | |
| Moose kidney | 2 | 7.31 | 4.09 | 7.31 | 4.41 | 10.20 |
| Moose liver | 2 | 1.54 | 0.39 | 1.54 | 1.26 | 1.81 |
| Deer liver | 1 | 0.32 | - | 0.32 | 0.32 | 0.32 |
| Yew bark | 1 | 0.31 | - | 0.31 | 0.31 | 0.31 |
| Devils club bark | 1 | 0.26 | - | 0.26 | 0.26 | 0.26 |
| **Taiga Plains** | | | | | | |
| Moose kidney | 2 | 16.39 | 15.01 | 16.39 | 5.77 | 27.00 |
| Rabbit/Hare liver | 1 | 3.75 | - | 3.75 | 3.75 | 3.75 |
| Moose liver | 2 | 1.67 | 1.31 | 1.67 | 0.74 | 2.60 |
| Moose heart | 2 | 1.45 | 2.02 | 1.45 | 0.03 | 2.88 |
| Rabbit/Hare meat | 3 | 0.81 | 1.38 | 0.01 | 0.01 | 2.40 |
| **Boreal Plains** | | | | | | |
| Beaver kidney | 1 | 21.60 | - | 21.60 | 21.60 | 21.60 |
| Rabbit/Hare kidney | 1 | 11.30 | - | 11.30 | 11.30 | 11.30 |
| Moose kidney | 16 | 10.19 | 9.87 | 6.92 | 0.41 | 31.10 |
| Deer kidney | 2 | 5.62 | 0.71 | 5.62 | 5.12 | 6.12 |
| Beaver liver | 1 | 3.44 | - | 3.44 | 3.44 | 3.44 |
| **Prairies** | | | | | | |
| Moose kidney | 2 | 7.77 | 7.40 | 7.77 | 2.53 | 13.00 |
| Elk kidney | 1 | 2.13 | - | 2.13 | 2.13 | 2.13 |
| Deer kidney | 3 | 1.99 | 1.38 | 1.46 | 0.95 | 3.55 |
| Rabbit/Hare kidney | 1 | 1.38 | - | 1.38 | 1.38 | 1.38 |
| Moose liver | 3 | 1.01 | 1.22 | 0.49 | 0.14 | 2.40 |
| **Taiga Shield** | | | | | | |
| Moose kidney | 1 | 12.60 | - | 12.60 | 12.60 | 12.60 |
| Caribou kidney | 3 | 3.89 | 3.40 | 5.23 | 0.02 | 6.42 |
| Moose liver | 1 | 0.72 | - | 0.72 | 0.72 | 0.72 |
| Caribou liver | 2 | 0.71 | 0.31 | 0.71 | 0.49 | 0.93 |
| Ptarmigan meat | 1 | 0.36 | - | 0.36 | 0.36 | 0.36 |
| **Boreal Shield** | | | | | | |
| Moose kidney | 9 | 14.24 | 8.62 | 13.00 | 0.00 | 29.80 |
| Deer kidney | 2 | 4.44 | 6.21 | 4.44 | 0.05 | 8.83 |
| Caribou kidney | 1 | 3.91 | - | 3.91 | 3.91 | 3.91 |
| Moose liver | 14 | 2.12 | 1.56 | 1.92 | 0.01 | 6.80 |
| Sea snails | 1 | 1.47 | - | 1.47 | 1.47 | 1.47 |
| **Hudson Plains** | | | | | | |
| Moose kidney | 4 | 13.25 | 10.89 | 14.05 | 0.00 | 24.90 |
| Moose liver | 5 | 1.52 | 0.91 | 1.21 | 0.72 | 2.85 |
| Beaver meat | 4 | 0.62 | 1.21 | 0.01 | 0.01 | 2.43 |
| Moose meat | 7 | 0.05 | 0.10 | 0.00 | 0.00 | 0.28 |
| Northern pike/ Jackfish eggs | 1 | 0.04 | - | 0.04 | 0.04 | 0.04 |
| **Mixedwood Plains** | | | | | | |
| Deer kidney | 2 | 3.22 | 4.25 | 3.22 | 0.22 | 6.22 |
| Tobacco | 1 | 0.39 | - | 0.39 | 0.39 | 0.39 |
| Fiddleheads | 2 | 0.39 | 0.53 | 0.39 | 0.01 | 0.76 |
| Deer liver | 3 | 0.15 | 0.04 | 0.14 | 0.12 | 0.20 |
| Mushrooms | 1 | 0.13 | - | 0.13 | 0.13 | 0.13 |
| **Atlantic Maritime** | | | | | | |
| Moose kidney | 3 | 7.90 | 5.26 | 5.67 | 4.12 | 13.90 |
| Moose liver | 9 | 2.50 | 2.04 | 1.99 | 0.01 | 5.80 |
| Oysters | 3 | 1.28 | 0.30 | 1.37 | 0.95 | 1.52 |
| Rabbit/Hare liver | 1 | 1.09 | - | 1.09 | 1.09 | 1.09 |
| Moose heart | 4 | 1.04 | 2.04 | 0.03 | 0.01 | 4.10 |

**Table 2: Lead**

| **Sample** | **N**  **Sites** | **Mean (ug/g)** | **SD (ug/g)** | **Median (ug/g)** | **Minimum (ug/g)** | **Maximum (ug/g)** |
| --- | --- | --- | --- | --- | --- | --- |
| **Pacific Maritime** | | | | | | |
| Grouse meat | 2 | 18.25 | 25.81 | 18.25 | 0.00 | 36.50 |
| Deer meat | 8 | 1.03 | 2.03 | 0.05 | 0.00 | 5.63 |
| Cascara bark | 1 | 0.90 | - | 0.90 | 0.90 | 0.90 |
| Bear liver | 1 | 0.73 | - | 0.73 | 0.73 | 0.73 |
| Rabbit/Hare meat | 1 | 0.60 | - | 0.60 | 0.60 | 0.60 |
| **Boreal Cordillera** | | | | | | |
| Caribou weeds | 1 | 0.30 | - | 0.30 | 0.30 | 0.30 |
| Blueberries | 1 | 0.00 | - | 0.00 | 0.00 | 0.00 |
| Trout | 2 | 0.00 | 0.00 | 0.00 | 0.00 | 0.00 |
| Moose meat | 2 | 0.00 | 0.00 | 0.00 | 0.00 | 0.00 |
| Moose liver | 1 | 0.00 | - | 0.00 | 0.00 | 0.00 |
| **Montane Cordillera** | | | | | | |
| Deer meat | 5 | 2.81 | 6.20 | 0.04 | 0.00 | 13.90 |
| Devils club bark | 1 | 0.70 | - | 0.70 | 0.70 | 0.70 |
| Black bear meat | 2 | 0.57 | 0.81 | 0.57 | 0.00 | 1.14 |
| Moose kidney | 2 | 0.50 | 0.49 | 0.50 | 0.15 | 0.85 |
| Rabbit/Hare meat | 2 | 0.34 | 0.44 | 0.34 | 0.03 | 0.65 |
| **Taiga Plains** | | | | | | |
| Grouse meat | 3 | 2.63 | 4.44 | 0.12 | 0.01 | 7.75 |
| Goose meat | 2 | 1.33 | 1.87 | 1.33 | 0.00 | 2.65 |
| Beaver fat | 1 | 0.77 | - | 0.77 | 0.77 | 0.77 |
| Duck meat | 4 | 0.09 | 0.18 | 0.01 | 0.00 | 0.36 |
| Deer meat | 1 | 0.04 | - | 0.04 | 0.04 | 0.04 |
| **Boreal Plains** | | | | | | |
| Bison meat | 3 | 43.75 | 75.56 | 0.24 | 0.01 | 131.00 |
| Duck heart | 1 | 9.34 | - | 9.34 | 9.34 | 9.34 |
| Grouse meat | 20 | 4.15 | 13.53 | 0.09 | 0.00 | 60.60 |
| Beaver heart | 1 | 2.69 | - | 2.69 | 2.69 | 2.69 |
| Rabbit/Hare meat | 13 | 2.15 | 7.56 | 0.01 | 0.00 | 27.30 |
| **Prairies** | | | | | | |
| Rabbit/Hare meat | 7 | 23.74 | 61.41 | 0.21 | 0.02 | 163.00 |
| Deer meat | 8 | 3.52 | 9.57 | 0.09 | 0.00 | 27.20 |
| Grouse meat | 8 | 3.29 | 8.35 | 0.07 | 0.00 | 23.90 |
| Duck gizzard | 2 | 1.89 | 2.57 | 1.89 | 0.07 | 3.70 |
| Ling cod/ Mariah liver | 1 | 0.67 | - | 0.67 | 0.67 | 0.67 |
| **Taiga Shield** | | | | | | |
| Caribou heart | 3 | 1.83 | 3.16 | 0.01 | 0.00 | 5.48 |
| Muskrat meat | 1 | 1.79 | - | 1.79 | 1.79 | 1.79 |
| Grouse meat | 5 | 1.51 | 2.43 | 0.52 | 0.06 | 5.84 |
| Ptarmigan meat | 1 | 0.27 | - | 0.27 | 0.27 | 0.27 |
| Moose tongue | 1 | 0.16 | - | 0.16 | 0.16 | 0.16 |
| **Boreal Shield** | | | | | | |
| Grouse meat | 25 | 8.84 | 30.47 | 0.33 | 0.00 | 152.00 |
| Duck meat | 19 | 6.68 | 23.70 | 0.04 | 0.00 | 104.00 |
| Beaver meat | 12 | 4.50 | 14.22 | 0.01 | 0.00 | 49.49 |
| Black bear meat | 5 | 2.75 | 6.07 | 0.01 | 0.00 | 13.60 |
| Goose meat | 13 | 1.51 | 4.37 | 0.18 | 0.00 | 16.00 |
| **Hudson Plains** | | | | | | |
| Grouse meat | 4 | 0.36 | 0.47 | 0.21 | 0.00 | 1.01 |
| Duck meat | 10 | 0.24 | 0.42 | 0.08 | 0.00 | 1.31 |
| Goose meat | 7 | 0.21 | 0.30 | 0.06 | 0.00 | 0.76 |
| Moose liver | 5 | 0.09 | 0.16 | 0.01 | 0.00 | 0.37 |
| Moose meat | 7 | 0.07 | 0.16 | 0.01 | 0.00 | 0.42 |
| **Mixedwood Plains** | | | | | | |
| Deer meat | 6 | 7.35 | 17.18 | 0.10 | 0.00 | 42.40 |
| Deer liver | 3 | 1.79 | 3.08 | 0.02 | 0.01 | 5.35 |
| Mushrooms | 1 | 1.19 | - | 1.19 | 1.19 | 1.19 |
| Tobacco | 1 | 1.10 | - | 1.10 | 1.10 | 1.10 |
| Onions | 1 | 1.07 | - | 1.07 | 1.07 | 1.07 |
| **Atlantic Maritime** | | | | | | |
| Squirrel meat | 2 | 45.38 | 62.11 | 45.38 | 1.46 | 89.30 |
| Rabbit/Hare meat | 8 | 5.23 | 14.14 | 0.03 | 0.02 | 40.20 |
| Dandelion roots | 1 | 3.79 | - | 3.79 | 3.79 | 3.79 |
| Grouse meat | 12 | 2.10 | 6.62 | 0.06 | 0.01 | 23.10 |
| Deer meat | 11 | 1.17 | 3.66 | 0.01 | 0.00 | 12.20 |

**Table 3: Arsenic**

| **Sample** | **N**  **Sites** | **Mean (ug/g)** | **SD (ug/g)** | **Median (ug/g)** | **Minimum (ug/g)** | **Maximum (ug/g)** |
| --- | --- | --- | --- | --- | --- | --- |
| **Pacific Maritime** | | | | | | |
| Seaweed | 5 | 25.27 | 13.37 | 31.00 | 3.45 | 35.10 |
| Octopus | 1 | 9.07 | - | 9.07 | 9.07 | 9.07 |
| Prawns | 3 | 8.91 | 1.13 | 8.48 | 8.06 | 10.20 |
| Crabs | 6 | 7.49 | 4.04 | 6.57 | 3.48 | 12.80 |
| Sea cucumber | 1 | 5.13 | Na | 5.13 | 5.13 | 5.13 |
| **Boreal Cordillera** | | | | | | |
| Salmon | 2 | 0.61 | 0.05 | 0.61 | 0.57 | 0.64 |
| Caribou weeds | 1 | 0.30 | - | 0.30 | 0.30 | 0.30 |
| Trout | 2 | 0.07 | 0.02 | 0.07 | 0.05 | 0.08 |
| Moose liver | 1 | 0.06 | - | 0.06 | 0.06 | 0.06 |
| Blueberries | 1 | 0.00 | - | 0.00 | 0.00 | 0.00 |
| **Montane Cordillera** | | | | | | |
| Halibut | 1 | 3.37 | - | 3.37 | 3.37 | 3.37 |
| Ooligan grease | 1 | 2.04 | - | 2.04 | 2.04 | 2.04 |
| Salmon | 9 | 0.71 | 0.17 | 0.64 | 0.53 | 1.01 |
| Ling cod/ Mariah/Burbot | 2 | 0.49 | 0.63 | 0.49 | 0.04 | 0.93 |
| Salmon eggs | 4 | 0.29 | 0.09 | 0.30 | 0.18 | 0.38 |
| **Taiga Plains** | | | | | | |
| Sweetflag/ Muskrat root | 2 | 0.75 | 0.78 | 0.75 | 0.20 | 1.30 |
| Salmon | 1 | 0.53 | - | 0.53 | 0.53 | 0.53 |
| Mushrooms | 1 | 0.20 | - | 0.20 | 0.20 | 0.20 |
| Labrador tea | 1 | 0.10 | - | 0.10 | 0.10 | 0.10 |
| Poplar tree bark | 1 | 0.08 | - | 0.08 | 0.08 | 0.08 |
| **Boreal Plains** | | | | | | |
| Dandelion greens | 1 | 1.80 | - | 1.80 | 1.80 | 1.80 |
| Currants | 1 | 0.60 | - | 0.60 | 0.60 | 0.60 |
| Lambs quarters leaves and stems | 1 | 0.46 | - | 0.46 | 0.46 | 0.46 |
| Cattail tops and stems | 1 | 0.31 | - | 0.31 | 0.31 | 0.31 |
| Duck meat | 22 | 0.21 | 0.90 | 0.01 | 0.00 | 4.22 |
| **Prairies** | | | | | | |
| Blueberry leaves | 1 | 0.42 | - | 0.42 | 0.42 | 0.42 |
| Sweetflag/ Muskrat root | 1 | 0.28 | - | 0.28 | 0.28 | 0.28 |
| Rabbit/Hare meat | 7 | 0.22 | 0.57 | 0.00 | 0.00 | 1.50 |
| Ling cod/ Mariah liver | 1 | 0.14 | - | 0.14 | 0.14 | 0.14 |
| Duck gizzard | 2 | 0.12 | 0.07 | 0.12 | 0.07 | 0.17 |
| **Taiga Shield** | | | | | | |
| Atlantic salmon | 1 | 0.56 | - | 0.56 | 0.56 | 0.56 |
| Whitefish | 4 | 0.20 | 0.17 | 0.18 | 0.01 | 0.41 |
| Sucker | 2 | 0.11 | 0.00 | 0.11 | 0.11 | 0.11 |
| Ling cod/ Mariah/Burbot | 1 | 0.09 | - | 0.09 | 0.09 | 0.09 |
| Trout | 8 | 0.06 | 0.07 | 0.02 | 0.01 | 0.17 |
| **Boreal Shield** | | | | | | |
| Lobster | 2 | 8.11 | 1.67 | 8.11 | 6.93 | 9.29 |
| Sea snails | 1 | 3.31 | - | 3.31 | 3.31 | 3.31 |
| Cod | 2 | 2.97 | 2.47 | 2.97 | 1.22 | 4.72 |
| Mussels | 1 | 2.95 | - | 2.95 | 2.95 | 2.95 |
| Cod eggs | 1 | 2.50 | - | 2.50 | 2.50 | 2.50 |
| **Hudson Plains** | | | | | | |
| Cisco | 1 | 1.93 | - | 1.93 | 1.93 | 1.93 |
| Whitefish | 4 | 1.85 | 0.65 | 1.66 | 1.30 | 2.77 |
| Northern pike/ Jackfish eggs | 1 | 0.75 | - | 0.75 | 0.75 | 0.75 |
| Northern pike/ Jackfish | 4 | 0.73 | 0.91 | 0.38 | 0.11 | 2.04 |
| Trout | 3 | 0.58 | 0.40 | 0.54 | 0.21 | 1.00 |
| **Mixedwood Plains** | | | | | | |
| Sturgeon | 2 | 0.58 | 0.18 | 0.58 | 0.45 | 0.71 |
| Mushrooms | 1 | 0.54 | - | 0.54 | 0.54 | 0.54 |
| Smelt | 1 | 0.37 | - | 0.37 | 0.37 | 0.37 |
| Tobacco | 1 | 0.20 | - | 0.20 | 0.20 | 0.20 |
| Salmon | 2 | 0.19 | 0.21 | 0.19 | 0.04 | 0.33 |
| **Atlantic Maritime** | | | | | | |
| Perch | 1 | 11.90 | - | 11.90 | 11.90 | 11.90 |
| Crabs | 8 | 11.12 | 7.83 | 7.91 | 4.91 | 25.90 |
| Shad | 1 | 7.44 | - | 7.44 | 7.44 | 7.44 |
| Sole | 2 | 5.78 | 6.11 | 5.78 | 1.46 | 10.10 |
| Lobster | 10 | 5.28 | 3.60 | 4.10 | 1.61 | 13.80 |

**Table 4: Mercury**

| **Sample** | **N**  **Sites** | **Mean (ug/g)** | **SD (ug/g)** | **Median (ug/g)** | **Minimum (ug/g)** | **Maximum (ug/g)** |
| --- | --- | --- | --- | --- | --- | --- |
| **Pacific Maritime** | | | | | | |
| Mushrooms | 5 | 0.21 | 0.28 | 0.06 | 0.01 | 0.68 |
| Halibut | 5 | 0.19 | 0.12 | 0.17 | 0.02 | 0.33 |
| Rockfish | 6 | 0.17 | 0.13 | 0.16 | 0.01 | 0.38 |
| Trout | 6 | 0.09 | 0.11 | 0.04 | 0.00 | 0.28 |
| Cockles | 3 | 0.05 | 0.08 | 0.01 | 0.00 | 0.15 |
| **Boreal Cordillera** | | | | | | |
| Trout | 2 | 0.15 | 0.22 | 0.15 | 0.00 | 0.31 |
| Salmon | 2 | 0.03 | 0.01 | 0.03 | 0.03 | 0.04 |
| Caribou weeds | 1 | 0.02 | - | 0.02 | 0.02 | 0.02 |
| Moose liver | 1 | 0.01 | - | 0.01 | 0.01 | 0.01 |
| Blueberries | 1 | 0.00 | - | 0.00 | 0.00 | 0.00 |
| **Montane Cordillera** | | | | | | |
| Arctic char | 1 | 0.92 | - | 0.92 | 0.92 | 0.92 |
| Carp | 1 | 0.72 | - | 0.72 | 0.72 | 0.72 |
| Ling cod/ Mariah/Burbot | 2 | 0.27 | 0.23 | 0.27 | 0.11 | 0.43 |
| Halibut | 1 | 0.22 | - | 0.22 | 0.22 | 0.22 |
| Groundhog meat | 1 | 0.09 | - | 0.09 | 0.09 | 0.09 |
| **Taiga Plains** | | | | | | |
| Northern pike/ Jackfish | 2 | 0.20 | 0.04 | 0.20 | 0.18 | 0.23 |
| Walleye/ Pickerel | 1 | 0.16 | - | 0.16 | 0.16 | 0.16 |
| Trout | 2 | 0.10 | 0.06 | 0.10 | 0.05 | 0.14 |
| Salmon | 1 | 0.04 | - | 0.04 | 0.04 | 0.04 |
| Arctic grayling | 1 | 0.02 | - | 0.02 | 0.02 | 0.02 |
| **Boreal Plains** | | | | | | |
| Walleye/ Pickerel | 12 | 0.46 | 0.26 | 0.38 | 0.07 | 1.02 |
| Northern pike/ Jackfish | 10 | 0.44 | 0.26 | 0.36 | 0.18 | 0.96 |
| Mooneye/ Goldeye | 1 | 0.20 | - | 0.20 | 0.20 | 0.20 |
| Ling cod/ Mariah/Burbot | 2 | 0.18 | 0.05 | 0.18 | 0.14 | 0.22 |
| Arctic grayling | 1 | 0.17 | - | 0.17 | 0.17 | 0.17 |
| **Prairies** | | | | | | |
| Walleye/ Pickerel | 3 | 0.19 | 0.04 | 0.21 | 0.14 | 0.22 |
| Northern pike/ Jackfish | 4 | 0.15 | 0.12 | 0.14 | 0.04 | 0.28 |
| Whitefish | 4 | 0.14 | 0.13 | 0.14 | 0.01 | 0.28 |
| Perch | 1 | 0.09 | - | 0.09 | 0.09 | 0.09 |
| Duck gizzard | 2 | 0.04 | 0.04 | 0.04 | 0.02 | 0.07 |
| **Taiga Shield** | | | | | | |
| Caribou kidney | 3 | 0.57 | 0.49 | 0.80 | 0.01 | 0.91 |
| Walleye/ Pickerel | 2 | 0.43 | 0.09 | 0.43 | 0.36 | 0.49 |
| Trout | 8 | 0.36 | 0.17 | 0.40 | 0.10 | 0.58 |
| Ling cod/ Mariah/Burbot | 1 | 0.28 | - | 0.28 | 0.28 | 0.28 |
| Northern pike/ Jackfish | 4 | 0.25 | 0.13 | 0.21 | 0.14 | 0.44 |
| **Boreal Shield** | | | | | | |
| Harp seal meat | 1 | 1.06 | - | 1.06 | 1.06 | 1.06 |
| Caribou kidney | 1 | 0.65 | - | 0.65 | 0.65 | 0.65 |
| Northern pike/ Jackfish | 13 | 0.58 | 0.72 | 0.29 | 0.15 | 2.75 |
| Carp | 1 | 0.37 | - | 0.37 | 0.37 | 0.37 |
| Walleye/ Pickerel | 21 | 0.37 | 0.29 | 0.28 | 0.08 | 1.27 |
| **Hudson Plains** | | | | | | |
| Northern pike/ Jackfish | 4 | 0.54 | 0.15 | 0.51 | 0.42 | 0.74 |
| Walleye/ Pickerel | 4 | 0.40 | 0.14 | 0.43 | 0.22 | 0.52 |
| Sturgeon | 4 | 0.39 | 0.19 | 0.35 | 0.20 | 0.63 |
| Trout | 3 | 0.12 | 0.01 | 0.12 | 0.11 | 0.14 |
| Whitefish | 4 | 0.10 | 0.03 | 0.10 | 0.07 | 0.12 |
| **Mixedwood Plains** | | | | | | |
| Mushrooms | 1 | 1.72 | - | 1.72 | 1.72 | 1.72 |
| Sturgeon | 2 | 0.40 | 0.23 | 0.40 | 0.24 | 0.56 |
| Walleye/ Pickerel | 6 | 0.39 | 0.21 | 0.36 | 0.18 | 0.78 |
| Bass | 4 | 0.38 | 0.23 | 0.37 | 0.11 | 0.66 |
| Trout | 3 | 0.21 | 0.06 | 0.19 | 0.16 | 0.28 |
| **Atlantic Maritime** | | | | | | |
| Bass | 4 | 0.47 | 0.43 | 0.33 | 0.14 | 1.07 |
| Striped bass | 7 | 0.16 | 0.09 | 0.12 | 0.03 | 0.32 |
| Sucker | 1 | 0.14 | - | 0.14 | 0.14 | 0.14 |
| Halibut | 3 | 0.14 | 0.12 | 0.11 | 0.03 | 0.26 |
| Eel | 9 | 0.11 | 0.03 | 0.12 | 0.06 | 0.14 |

**Table 5: Methyl Mercury**

| **Sample** | **N**  **Sites** | **Mean (ug/g)** | **SD (ug/g)** | **Median (ug/g)** | **Minimum (ug/g)** | **Maximum (ug/g)** |
| --- | --- | --- | --- | --- | --- | --- |
| **Pacific Maritime** | | | | | | |
| Halibut | 5 | 0.27 | 0.08 | 0.28 | 0.18 | 0.38 |
| Rockfish | 6 | 0.24 | 0.13 | 0.19 | 0.11 | 0.41 |
| Trout | 6 | 0.14 | 0.12 | 0.10 | 0.03 | 0.36 |
| Cod | 2 | 0.07 | 0.01 | 0.07 | 0.06 | 0.08 |
| Crabs | 6 | 0.06 | 0.04 | 0.04 | 0.03 | 0.13 |
| **Boreal Cordillera** | | | | | | |
| Trout | 2 | 0.11 | 0.02 | 0.11 | 0.10 | 0.12 |
| Salmon | 2 | 0.04 | 0.00 | 0.04 | 0.03 | 0.04 |
| Moose meat | 2 | 0.00 | 0.00 | 0.00 | 0.00 | 0.00 |
| Moose liver | 1 | 0.00 | Na | 0.00 | 0.00 | 0.00 |
| **Montane Cordillera** | | | | | | |
| Arctic char | 1 | 0.74 | - | 0.74 | 0.74 | 0.74 |
| Ling cod/ Mariah/Burbot | 1 | 0.36 | - | 0.36 | 0.36 | 0.36 |
| Carp | 1 | 0.18 | - | 0.18 | 0.18 | 0.18 |
| Halibut | 1 | 0.17 | - | 0.17 | 0.17 | 0.17 |
| Trout | 6 | 0.17 | 0.19 | 0.10 | 0.06 | 0.54 |
| **Taiga Plains** | | | | | | |
| Walleye/ Pickerel | 1 | 0.32 | - | 0.32 | 0.32 | 0.32 |
| Northern pike/ Jackfish | 2 | 0.15 | 0.03 | 0.15 | 0.13 | 0.17 |
| Trout | 2 | 0.12 | 0.05 | 0.12 | 0.08 | 0.15 |
| Salmon | 1 | 0.05 | - | 0.05 | 0.05 | 0.05 |
| Duck meat | 1 | 0.01 | - | 0.01 | 0.01 | 0.01 |
| **Boreal Plains** | | | | | | |
| Northern pike/ Jackfish | 10 | 0.27 | 0.16 | 0.27 | 0.08 | 0.58 |
| Walleye/ Pickerel | 12 | 0.27 | 0.19 | 0.28 | 0.03 | 0.67 |
| Trout | 9 | 0.18 | 0.24 | 0.04 | 0.01 | 0.69 |
| Ling cod/ Mariah/Burbot | 1 | 0.13 | - | 0.13 | 0.13 | 0.13 |
| Sucker | 4 | 0.06 | 0.03 | 0.06 | 0.04 | 0.08 |
| **Prairies** | | | | | | |
| Walleye/ Pickerel | 3 | 0.17 | 0.06 | 0.15 | 0.12 | 0.24 |
| Northern pike/ Jackfish | 4 | 0.10 | 0.07 | 0.09 | 0.04 | 0.18 |
| Whitefish | 4 | 0.10 | 0.14 | 0.03 | 0.01 | 0.30 |
| Perch | 1 | 0.08 | - | 0.08 | 0.08 | 0.08 |
| Duck gizzard | 2 | 0.06 | 0.04 | 0.06 | 0.03 | 0.09 |
| **Taiga Shield** | | | | | | |
| Trout | 8 | 0.44 | 0.26 | 0.44 | 0.14 | 0.95 |
| Walleye/ Pickerel | 2 | 0.42 | 0.07 | 0.42 | 0.37 | 0.47 |
| Ling cod/ Mariah/Burbot | 1 | 0.36 | - | 0.36 | 0.36 | 0.36 |
| Duck meat | 3 | 0.24 | 0.16 | 0.16 | 0.13 | 0.42 |
| Northern pike/ Jackfish | 4 | 0.22 | 0.19 | 0.15 | 0.09 | 0.49 |
| **Boreal Shield** | | | | | | |
| Harp seal meat | 1 | 1.39 | - | 1.39 | 1.39 | 1.39 |
| Walleye/ Pickerel | 14 | 0.38 | 0.48 | 0.16 | 0.06 | 1.49 |
| Northern pike/ Jackfish | 10 | 0.36 | 0.24 | 0.28 | 0.08 | 0.72 |
| Lobster | 2 | 0.32 | 0.23 | 0.32 | 0.16 | 0.49 |
| Trout | 15 | 0.28 | 0.23 | 0.29 | 0.03 | 0.90 |
| **Hudson Plains** | | | | | | |
| Northern pike/ Jackfish | 4 | 0.33 | 0.22 | 0.29 | 0.15 | 0.61 |
| Sturgeon | 4 | 0.27 | 0.20 | 0.23 | 0.09 | 0.54 |
| Walleye/ Pickerel | 3 | 0.25 | 0.24 | 0.14 | 0.09 | 0.53 |
| Trout | 3 | 0.09 | 0.04 | 0.07 | 0.06 | 0.14 |
| Whitefish | 4 | 0.06 | 0.01 | 0.06 | 0.04 | 0.07 |
| **Mixedwood Plains** | | | | | | |
| Walleye/ Pickerel | 6 | 0.21 | 0.20 | 0.10 | 0.04 | 0.49 |
| Bass | 3 | 0.19 | 0.12 | 0.26 | 0.05 | 0.27 |
| Sturgeon | 2 | 0.19 | 0.06 | 0.19 | 0.15 | 0.23 |
| Trout | 3 | 0.17 | 0.16 | 0.07 | 0.07 | 0.36 |
| Catfish | 3 | 0.10 | 0.05 | 0.08 | 0.06 | 0.16 |
| **Atlantic Maritime** | | | | | | |
| Bass | 3 | 0.60 | 0.80 | 0.14 | 0.13 | 1.53 |
| Sucker | 1 | 0.14 | - | 0.14 | 0.14 | 0.14 |
| Striped bass | 6 | 0.13 | 0.10 | 0.10 | 0.03 | 0.32 |
| Eel | 8 | 0.10 | 0.04 | 0.11 | 0.04 | 0.16 |
| Crabs | 2 | 0.10 | 0.10 | 0.10 | 0.02 | 0.17 |

**Table 6: DDE**

| **Sample** | **N**  **Sites** | **Mean (ng/g)** | **SD (ng/g)** | **Median (ng/g)** | **Minimum (ng/g)** | **Maximum (ng/g)** |
| --- | --- | --- | --- | --- | --- | --- |
| **Pacific Maritime** | | | | | | |
| Ooligan grease | 4 | 22.65 | 6.00 | 21.90 | 16.50 | 30.30 |
| Salmon | 37 | 3.25 | 3.73 | 2.41 | 0.00 | 21.20 |
| Cod | 2 | 2.56 | 2.28 | 2.56 | 0.94 | 4.17 |
| Ooligan | 4 | 2.54 | 1.40 | 2.46 | 1.12 | 4.10 |
| Salmon eggs | 6 | 2.31 | 1.19 | 2.27 | 0.80 | 4.38 |
| **Boreal Cordillera** | | | | | | |
| Salmon | 2 | 0.87 | 1.22 | 0.87 | 0.00 | 1.73 |
| Blueberries | 1 | 0.00 | - | 0.00 | 0.00 | 0.00 |
| Trout | 2 | 0.00 | 0.00 | 0.00 | 0.00 | 0.00 |
| Moose meat | 2 | 0.00 | 0.00 | 0.00 | 0.00 | 0.00 |
| Moose liver | 1 | 0.00 | - | 0.00 | 0.00 | 0.00 |
| **Montane Cordillera** | | | | | | |
| Ooligan grease | 1 | 15.00 | - | 15.00 | 15.00 | 15.00 |
| Trout | 6 | 5.33 | 9.90 | 0.40 | 0.00 | 24.90 |
| Ling cod/ Mariah/Burbot | 2 | 2.77 | 3.91 | 2.77 | 0.00 | 5.53 |
| Salmon eggs | 4 | 2.14 | 4.27 | 0.00 | 0.00 | 8.54 |
| Salmon | 9 | 1.59 | 0.77 | 1.76 | 0.00 | 2.36 |
| **Taiga Plains** | | | | | | |
| Goose meat | 1 | 4.96 | - | 4.96 | 4.96 | 4.96 |
| Salmon | 1 | 3.71 | - | 3.71 | 3.71 | 3.71 |
| Duck meat | 1 | 1.24 | - | 1.24 | 1.24 | 1.24 |
| Arctic grayling | 1 | 0.70 | - | 0.70 | 0.70 | 0.70 |
| Northern pike/ Jackfish | 2 | 0.03 | 0.04 | 0.03 | 0.00 | 0.06 |
| **Boreal Plains** | | | | | | |
| Beaver kidney | 1 | 16.10 | - | 16.10 | 16.10 | 16.10 |
| Beaver liver | 1 | 13.80 | - | 13.80 | 13.80 | 13.80 |
| Elk liver | 1 | 9.39 | - | 9.39 | 9.39 | 9.39 |
| Trout | 9 | 6.15 | 10.53 | 1.66 | 0.00 | 32.50 |
| Beaver meat | 3 | 5.04 | 4.22 | 3.78 | 1.59 | 9.75 |
| **Prairies** | | | | | | |
| Deer liver | 2 | 5.75 | 8.13 | 5.75 | 0.00 | 11.50 |
| Whitefish | 4 | 1.99 | 2.50 | 0.97 | 0.33 | 5.68 |
| Duck meat | 5 | 1.20 | 0.75 | 1.57 | 0.06 | 1.93 |
| Walleye/ Pickerel | 3 | 0.19 | 0.32 | 0.00 | 0.00 | 0.56 |
| Northern pike/ Jackfish | 4 | 0.05 | 0.08 | 0.02 | 0.00 | 0.17 |
| **Taiga Shield** | | | | | | |
| Duck meat | 1 | 102.00 | - | 102.00 | 102.00 | 102.00 |
| Trout | 7 | 5.83 | 4.87 | 5.19 | 1.37 | 15.70 |
| Whitefish | 4 | 1.28 | 0.82 | 1.31 | 0.24 | 2.25 |
| Trout eggs | 2 | 0.83 | 0.37 | 0.83 | 0.57 | 1.09 |
| Goose liver | 1 | 0.31 | - | 0.31 | 0.31 | 0.31 |
| **Boreal Shield** | | | | | | |
| Salmon eggs | 1 | 64.30 | - | 64.30 | 64.30 | 64.30 |
| Harp seal meat | 1 | 28.50 | - | 28.50 | 28.50 | 28.50 |
| Salmon | 5 | 24.13 | 23.76 | 12.40 | 5.89 | 61.10 |
| Duck meat | 8 | 13.53 | 27.44 | 5.22 | 0.00 | 81.00 |
| Trout | 18 | 12.15 | 17.46 | 4.82 | 0.33 | 64.95 |
| **Hudson Plains** | | | | | | |
| Goose meat | 6 | 14.13 | 15.41 | 9.37 | 1.66 | 42.90 |
| Duck meat | 1 | 5.04 | - | 5.04 | 5.04 | 5.04 |
| Black bear Fat | 1 | 3.39 | - | 3.39 | 3.39 | 3.39 |
| Sturgeon | 4 | 2.90 | 2.70 | 2.00 | 0.77 | 6.84 |
| Whitefish eggs | 1 | 2.13 | - | 2.13 | 2.13 | 2.13 |
| **Mixedwood Plains** | | | | | | |
| Trout | 3 | 70.93 | 59.97 | 102.00 | 1.80 | 109.00 |
| Smelt | 1 | 28.35 | - | 28.35 | 28.35 | 28.35 |
| Salmon | 2 | 25.65 | 23.13 | 25.65 | 9.29 | 42.00 |
| Sturgeon | 2 | 22.30 | 5.52 | 22.30 | 18.40 | 26.20 |
| Catfish | 3 | 10.90 | 7.21 | 13.70 | 2.71 | 16.30 |
| **Atlantic Maritime** | | | | | | |
| Bass | 3 | 19.13 | 30.12 | 2.43 | 1.05 | 53.90 |
| Eel | 7 | 9.66 | 11.89 | 4.53 | 1.10 | 35.10 |
| Trout | 19 | 6.73 | 10.53 | 2.23 | 0.51 | 38.50 |
| Atlantic salmon | 12 | 5.59 | 3.50 | 4.98 | 1.59 | 11.70 |
| Shad | 1 | 4.54 | - | 4.54 | 4.54 | 4.54 |

**Table 7: PCBs**

| **Sample** | **N**  **Sites** | **Mean (ng/g)** | **SD (ng/g)** | **Median (ng/g)** | **Minimum (ng/g)** | **Maximum (ng/g)** |
| --- | --- | --- | --- | --- | --- | --- |
| **Pacific Maritime** | | | | | | |
| Pacific herring | 1 | 8.24 | - | 8.24 | 8.24 | 8.24 |
| Prawns | 3 | 1.39 | 2.40 | 0.00 | 0.00 | 4.16 |
| Ooligan grease | 4 | 1.11 | 2.23 | 0.00 | 0.00 | 4.45 |
| Trout | 6 | 1.04 | 1.19 | 0.87 | 0.00 | 2.70 |
| Halibut | 5 | 0.87 | 1.12 | 0.46 | 0.00 | 2.67 |
| **Boreal Cordillera** | | | | | | |
| Blueberries | 1 | 0.00 | - | 0.00 | 0.00 | 0.00 |
| Trout | 2 | 0.00 | 0.00 | 0.00 | 0.00 | 0.00 |
| Moose meat | 2 | 0.00 | 0.00 | 0.00 | 0.00 | 0.00 |
| Moose liver | 1 | 0.00 | - | 0.00 | 0.00 | 0.00 |
| Black bear Fat | 1 | 0.00 | - | 0.00 | 0.00 | 0.00 |
| **Montane Cordillera** | | | | | | |
| Arctic char | 1 | 1.63 | - | 1.63 | 1.63 | 1.63 |
| Salmon eggs | 4 | 1.20 | 2.40 | 0.00 | 0.00 | 4.79 |
| Ling cod/ Mariah/Burbot | 2 | 0.23 | 0.32 | 0.23 | 0.00 | 0.45 |
| Trout | 6 | 0.14 | 0.22 | 0.00 | 0.00 | 0.47 |
| Salmon | 9 | 0.14 | 0.21 | 0.00 | 0.00 | 0.44 |
| **Taiga Plains** | | | | | | |
| Salmon | 1 | 1.14 | - | 1.14 | 1.14 | 1.14 |
| Trout | 2 | 0.78 | 1.10 | 0.78 | 0.00 | 1.55 |
| Northern pike/ Jackfish | 2 | 0.00 | 0.00 | 0.00 | 0.00 | 0.00 |
| Walleye/ Pickerel | 1 | 0.00 | - | 0.00 | 0.00 | 0.00 |
| Beaver meat | 2 | 0.00 | 0.00 | 0.00 | 0.00 | 0.00 |
| **Boreal Plains** | | | | | | |
| Duck meat | 7 | 24.34 | 64.21 | 0.00 | 0.00 | 169.95 |
| Elk liver | 1 | 10.72 | - | 10.72 | 10.72 | 10.72 |
| Beaver meat | 3 | 4.95 | 4.15 | 5.43 | 0.58 | 8.83 |
| Trout | 9 | 2.65 | 4.28 | 0.41 | 0.00 | 12.32 |
| Rabbit/Hare meat | 2 | 0.35 | 0.49 | 0.35 | 0.00 | 0.69 |
| **Prairies** | | | | | | |
| Whitefish | 4 | 1.46 | 2.19 | 0.56 | 0.00 | 4.71 |
| Deer liver | 2 | 0.55 | 0.78 | 0.55 | 0.00 | 1.10 |
| Walleye/ Pickerel | 3 | 0.30 | 0.51 | 0.00 | 0.00 | 0.89 |
| Duck meat | 5 | 0.28 | 0.38 | 0.00 | 0.00 | 0.75 |
| Perch | 1 | 0.00 | - | 0.00 | 0.00 | 0.00 |
| **Taiga Shield** | | | | | | |
| Duck meat | 1 | 127.71 | - | 127.71 | 127.71 | 127.71 |
| Black bear Fat | 1 | 19.63 | - | 19.63 | 19.63 | 19.63 |
| Trout | 7 | 6.53 | 5.38 | 4.89 | 0.00 | 15.18 |
| Whitefish | 4 | 1.97 | 2.16 | 1.44 | 0.19 | 4.80 |
| Trout eggs | 2 | 0.67 | 0.94 | 0.67 | 0.00 | 1.33 |
| **Boreal Shield** | | | | | | |
| Harp seal meat | 1 | 265.40 | - | 265.40 | 265.40 | 265.40 |
| Carp | 1 | 126.52 | - | 126.52 | 126.52 | 126.52 |
| Salmon eggs | 1 | 111.34 | - | 111.34 | 111.34 | 111.34 |
| Duck meat | 8 | 84.12 | 201.65 | 11.12 | 0.00 | 582.01 |
| Salmon | 5 | 67.51 | 62.07 | 36.44 | 18.31 | 161.20 |
| **Hudson Plains** | | | | | | |
| Black bear Fat | 1 | 7.13 | - | 7.13 | 7.13 | 7.13 |
| Northern pike/ Jackfish eggs | 1 | 4.76 | - | 4.76 | 4.76 | 4.76 |
| Whitefish eggs | 1 | 4.29 | - | 4.29 | 4.29 | 4.29 |
| Sturgeon | 4 | 3.44 | 2.56 | 3.72 | 0.56 | 5.78 |
| Northern pike/ Jackfish | 4 | 1.88 | 1.60 | 1.54 | 0.46 | 3.98 |
| **Mixedwood Plains** | | | | | | |
| Sturgeon | 2 | 324.00 | 39.53 | 324.00 | 296.04 | 351.95 |
| Trout | 3 | 194.16 | 166.65 | 282.01 | 1.96 | 298.51 |
| Catfish | 3 | 110.63 | 111.34 | 89.06 | 11.65 | 231.17 |
| Salmon | 2 | 73.83 | 43.35 | 73.83 | 43.18 | 104.48 |
| Smelt | 1 | 64.47 | - | 64.47 | 64.47 | 64.47 |
| **Atlantic Maritime** | | | | | | |
| Bass | 2 | 21.30 | 26.27 | 21.30 | 2.73 | 39.88 |
| Eel | 7 | 9.01 | 10.42 | 5.73 | 1.83 | 31.61 |
| Trout | 19 | 8.13 | 12.57 | 3.05 | 0.21 | 45.57 |
| Mackerel | 7 | 7.82 | 3.62 | 7.21 | 3.28 | 13.39 |
| Atlantic salmon | 11 | 6.75 | 4.36 | 4.42 | 2.81 | 15.36 |
