# Supplementary material for: Levels of metals and persistent organic pollutants in traditional foods consumed by First Nations living on-reserve in Canada
Source: Can J Public Health. 2021 Jun 28;112(Suppl 1):81–96. doi: 10.17269/s41997-021-00495-7 (PMC8239065; doi:10.17269/s41997-021-00495-7)
Supplement: Supplementary file 4 — (DOCX 60 kb) [file 41997_2021_495_MOESM4_ESM.docx]

**Supplementary Material D**

**Main Contributors to Contaminant Intake in Consumers of Traditional Foods by Ecozone**

**Table 1: Cadmium**

| Sample | Mean (ug/day) | Lower 95% CI (ug/day) | Upper 95% CI (ug/day) |
| --- | --- | --- | --- |
| Pacific Maritime | | | |
| Oysters | 2.45 | 0.00 | 6.26 |
| Seaweed | 1.23 | 0.04 | 2.43 |
| Moose liver | 0.82 | 0.19 | 1.46 |
| Mussels | 0.74 | 0.53 | 0.94 |
| Herring eggs | 0.28 | 0.05 | 0.51 |
| Boreal Cordillera | | | |
| Moose liver | 20.50 | 2.48 | 38.51 |
| Moose meat | 0.98 | 0.54 | 1.43 |
| Salmon | 0.04 | 0.03 | 0.04 |
| Blueberries | 0.00 | 0.00 | 0.00 |
| Trout | 0.00 | 0.00 | 0.00 |
| Montane Cordillera | | | |
| Moose kidney | 5.41 | 0.00 | 11.42 |
| Moose liver | 1.55 | 0.00 | 3.37 |
| Deer liver | 0.68 | 0.00 | 1.47 |
| Moose meat | 0.20 | 0.13 | 0.27 |
| Deer meat | 0.08 | 0.01 | 0.15 |
| Taiga Plains | | | |
| Moose kidney | 13.62 | 4.14 | 23.09 |
| Moose liver | 2.68 | 0.67 | 4.69 |
| Grouse meat | 0.53 | 0.26 | 0.80 |
| Moose meat | 0.52 | 0.36 | 0.68 |
| Walleye/Pickerel | 0.03 | 0.00 | 0.06 |
| Boreal Plains | | | |
| Moose kidney | 9.46 | 2.01 | 16.92 |
| Moose liver | 2.07 | 0.52 | 3.62 |
| Deer kidney | 0.84 | 0.00 | 1.76 |
| Moose meat | 0.11 | 0.04 | 0.18 |
| Deer liver | 0.04 | 0.00 | 0.08 |
| Prairies | | | |
| Moose kidney | 0.84 | 0.00 | 1.69 |
| Deer kidney | 0.54 | 0.00 | 1.32 |
| Elk kidney | 0.24 | 0.01 | 0.48 |
| Moose liver | 0.13 | 0.00 | 0.29 |
| Deer liver | 0.12 | 0.03 | 0.21 |
| Boreal Shield | | | |
| Moose kidney | 7.04 | 1.55 | 12.53 |
| Moose liver | 1.63 | 0.42 | 2.83 |
| Mussels | 0.92 | 0.00 | 2.69 |
| Caribou kidney | 0.62 | 0.00 | 1.38 |
| Rabbit/Hare heart | 0.17 | 0.09 | 0.26 |
| Taiga Shield | | | |
| Caribou kidney | 3.41 | 2.04 | 4.78 |
| Ptarmigan meat | 1.90 | 0.00 | 4.57 |
| Moose kidney | 0.45 | 0.11 | 0.80 |
| Caribou liver | 0.30 | 0.03 | 0.57 |
| Caribou meat | 0.19 | 0.08 | 0.30 |
| Hudson Plains | | | |
| Moose kidney | 4.75 | 2.93 | 6.57 |
| Beaver meat | 0.57 | 0.22 | 0.93 |
| Moose meat | 0.53 | 0.32 | 0.75 |
| Moose liver | 0.47 | 0.26 | 0.69 |
| Ptarmigan meat | 0.03 | 0.01 | 0.04 |
| Mixedwood Plains | | | |
| Fiddleheads | 0.04 | 0.00 | 0.07 |
| Deer meat | 0.03 | 0.01 | 0.05 |
| Strawberries | 0.02 | 0.02 | 0.03 |
| Deer kidney | 0.01 | 0.00 | 0.03 |
| Moose meat | 0.01 | 0.01 | 0.01 |
| Atlantic Maritime | | | |
| Lobster | 0.60 | 0.50 | 0.70 |
| Oysters | 0.30 | 0.18 | 0.43 |
| Mussels | 0.12 | 0.08 | 0.16 |
| Scallops | 0.11 | 0.06 | 0.16 |
| Moose kidney | 0.10 | 0.02 | 0.18 |

**Table 2: Lead**

| Sample | Mean (ug/day) | Lower 95% CI (ug/day) | Upper 95% CI (ug/day) |
| --- | --- | --- | --- |
| Pacific Maritime | | | |
| Deer meat | 3.56 | 0.00 | 7.14 |
| Grouse meat | 1.58 | 0.29 | 2.88 |
| Halibut | 1.00 | 0.43 | 1.58 |
| Elk meat | 0.12 | 0.00 | 0.28 |
| Seaweed | 0.10 | 0.00 | 0.19 |
| Boreal Cordillera | | | |
| Blueberries | 0.00 | 0.00 | 0.00 |
| Trout | 0.00 | 0.00 | 0.00 |
| Moose meat | 0.00 | 0.00 | 0.00 |
| Moose liver | 0.00 | 0.00 | 0.00 |
| Salmon | 0.00 | 0.00 | 0.00 |
| Montane Cordillera | | | |
| Deer meat | 51.15 | 3.40 | 98.91 |
| Moose kidney | 0.37 | 0.00 | 0.78 |
| Moose meat | 0.20 | 0.13 | 0.26 |
| Black bear meat | 0.12 | 0.01 | 0.22 |
| Grouse meat | 0.08 | 0.00 | 0.17 |
| Taiga Plains | | | |
| Grouse meat | 11.33 | 5.50 | 17.16 |
| Goose meat | 3.46 | 2.09 | 4.82 |
| Duck meat | 0.20 | 0.02 | 0.38 |
| Moose meat | 0.10 | 0.07 | 0.13 |
| Deer meat | 0.04 | 0.01 | 0.06 |
| Boreal Plains | | | |
| Bison meat | 8.81 | 0.37 | 17.25 |
| Moose meat | 8.65 | 3.34 | 13.96 |
| Deer meat | 2.21 | 0.75 | 3.67 |
| Grouse meat | 1.93 | 0.68 | 3.19 |
| Elk meat | 0.99 | 0.45 | 1.54 |
| Prairies | | | |
| Deer meat | 13.93 | 9.43 | 18.43 |
| Grouse meat | 0.66 | 0.00 | 1.54 |
| Goose meat | 0.15 | 0.00 | 0.42 |
| Moose meat | 0.10 | 0.06 | 0.14 |
| Duck meat | 0.05 | 0.00 | 0.12 |
| Boreal Shield | | | |
| Moose meat | 6.61 | 4.73 | 8.48 |
| Grouse meat | 4.82 | 2.51 | 7.12 |
| Beaver meat | 3.52 | 1.09 | 5.95 |
| Goose meat | 2.28 | 0.36 | 4.21 |
| Duck meat | 1.64 | 0.00 | 3.61 |
| Taiga Shield | | | |
| Grouse meat | 2.78 | 2.27 | 3.28 |
| Caribou heart | 2.50 | 1.72 | 3.28 |
| Ptarmigan meat | 1.40 | 0.00 | 3.36 |
| Caribou meat | 1.03 | 0.41 | 1.64 |
| Caribou kidney | 0.11 | 0.07 | 0.16 |
| Hudson Plains | | | |
| Goose meat | 1.71 | 1.04 | 2.38 |
| Moose meat | 0.73 | 0.43 | 1.02 |
| Grouse meat | 0.15 | 0.02 | 0.29 |
| Northern pike/Jackfish | 0.05 | 0.01 | 0.09 |
| Duck meat | 0.05 | 0.03 | 0.07 |
| Mixedwood Plains | | | |
| Deer meat | 28.99 | 6.51 | 51.46 |
| Moose meat | 0.32 | 0.22 | 0.42 |
| Strawberries | 0.17 | 0.11 | 0.22 |
| Deer liver | 0.09 | 0.00 | 0.24 |
| Wild ginger | 0.03 | 0.00 | 0.09 |
| Atlantic Maritime | | | |
| Deer meat | 1.45 | 1.00 | 1.91 |
| Moose meat | 0.31 | 0.14 | 0.47 |
| Squirrel meat | 0.09 | 0.01 | 0.17 |
| Mussels | 0.09 | 0.06 | 0.12 |
| Shrimp | 0.06 | 0.04 | 0.08 |

**Table 3: Arsenic**

| Sample | Mean (ug/day) | Lower 95% CI (ug/day) | Upper 95% CI (ug/day) |
| --- | --- | --- | --- |
| Pacific Maritime | | | |
| Prawns | 18.28 | 0.00 | 37.49 |
| Halibut | 12.40 | 5.32 | 19.49 |
| Seaweed | 7.81 | 0.23 | 15.40 |
| Clams | 7.58 | 2.51 | 12.65 |
| Ooligan grease | 5.93 | 0.36 | 11.50 |
| Boreal Cordillera | | | |
| Salmon | 1.98 | 1.75 | 2.21 |
| Moose liver | 0.15 | 0.02 | 0.27 |
| Trout | 0.03 | 0.00 | 0.05 |
| Blueberries | 0.00 | 0.00 | 0.00 |
| Moose meat | 0.00 | 0.00 | 0.00 |
| Montane Cordillera | | | |
| Salmon | 1.69 | 1.07 | 2.30 |
| Halibut | 0.95 | 0.00 | 1.96 |
| Deer meat | 0.51 | 0.03 | 0.99 |
| Salmon eggs | 0.42 | 0.02 | 0.81 |
| Ling cod/Mariah/Burbot | 0.22 | 0.00 | 0.59 |
| Taiga Plains | | | |
| Moose meat | 0.41 | 0.28 | 0.53 |
| Northern pike/Jackfish | 0.25 | 0.07 | 0.44 |
| Salmon | 0.17 | 0.08 | 0.27 |
| Beaver meat | 0.13 | 0.06 | 0.19 |
| Walleye/Pickerel | 0.11 | 0.00 | 0.26 |
| Boreal Plains | | | |
| Moose meat | 0.13 | 0.05 | 0.21 |
| Walleye/Pickerel | 0.09 | 0.05 | 0.14 |
| Northern pike/Jackfish | 0.05 | 0.03 | 0.08 |
| Dandelion greens | 0.03 | 0.00 | 0.07 |
| Whitefish | 0.03 | 0.01 | 0.04 |
| Prairies | | | |
| Walleye/Pickerel | 0.09 | 0.00 | 0.18 |
| Northern pike/Jackfish | 0.03 | 0.01 | 0.04 |
| Deer meat | 0.02 | 0.02 | 0.03 |
| Whitefish | 0.02 | 0.01 | 0.03 |
| Moose meat | 0.01 | 0.01 | 0.02 |
| Boreal Shield | | | |
| Mussels | 3.98 | 0.00 | 11.60 |
| Lobster | 1.15 | 0.90 | 1.39 |
| Cod | 0.75 | 0.58 | 0.92 |
| Walleye/Pickerel | 0.66 | 0.42 | 0.91 |
| Whitefish | 0.26 | 0.09 | 0.44 |
| Taiga Shield | | | |
| Whitefish | 0.53 | 0.06 | 1.00 |
| Caribou meat | 0.35 | 0.14 | 0.56 |
| Trout | 0.06 | 0.06 | 0.07 |
| Atlantic salmon | 0.04 | 0.00 | 0.11 |
| Northern pike/Jackfish | 0.04 | 0.01 | 0.06 |
| Hudson Plains | | | |
| Whitefish | 1.91 | 0.52 | 3.31 |
| Northern pike/Jackfish | 1.62 | 0.39 | 2.86 |
| Cisco | 1.12 | 0.67 | 1.56 |
| Walleye/Pickerel | 1.10 | 0.88 | 1.32 |
| Sturgeon | 0.24 | 0.16 | 0.31 |
| Mixedwood Plains | | | |
| Salmon | 0.10 | 0.00 | 0.22 |
| Walleye/Pickerel | 0.08 | 0.05 | 0.10 |
| Sturgeon | 0.08 | 0.00 | 0.15 |
| Perch | 0.05 | 0.02 | 0.09 |
| Maple syrup | 0.03 | 0.01 | 0.04 |
| Atlantic Maritime | | | |
| Lobster | 9.95 | 8.35 | 11.54 |
| Crabs | 3.28 | 2.23 | 4.33 |
| Shrimp | 2.72 | 1.85 | 3.59 |
| Haddock | 2.70 | 1.57 | 3.82 |
| Scallops | 1.48 | 0.86 | 2.10 |

**Table 4: Mercury**

| Sample | Mean (ug/day) | Lower 95% CI (ug/day) | Upper 95% CI (ug/day) |
| --- | --- | --- | --- |
| Pacific Maritime | | | |
| Halibut | 1.02 | 0.44 | 1.60 |
| Rockfish | 0.24 | 0.14 | 0.34 |
| Salmon | 0.12 | 0.08 | 0.17 |
| Salmon eggs | 0.07 | 0.03 | 0.10 |
| Cockles | 0.04 | 0.02 | 0.06 |
| Boreal Cordillera | | | |
| Salmon | 0.11 | 0.10 | 0.13 |
| Trout | 0.07 | 0.01 | 0.12 |
| Moose liver | 0.01 | 0.00 | 0.02 |
| Blueberries | 0.00 | 0.00 | 0.00 |
| Moose meat | 0.00 | 0.00 | 0.00 |
| Montane Cordillera | | | |
| Ling cod/Mariah/Burbot | 0.12 | 0.00 | 0.33 |
| Salmon eggs | 0.07 | 0.00 | 0.13 |
| Salmon | 0.07 | 0.04 | 0.09 |
| Halibut | 0.06 | 0.00 | 0.13 |
| Trout | 0.02 | 0.01 | 0.04 |
| Taiga Plains | | | |
| Northern pike/Jackfish | 1.42 | 0.41 | 2.44 |
| Walleye/Pickerel | 0.47 | 0.00 | 1.05 |
| Duck meat | 0.02 | 0.00 | 0.03 |
| Salmon | 0.01 | 0.01 | 0.02 |
| Moose kidney | 0.01 | 0.00 | 0.02 |
| Boreal Plains | | | |
| Walleye/Pickerel | 0.85 | 0.43 | 1.27 |
| Northern pike/Jackfish | 0.65 | 0.35 | 0.94 |
| Whitefish | 0.06 | 0.03 | 0.09 |
| Moose meat | 0.02 | 0.01 | 0.03 |
| Moose kidney | 0.01 | 0.00 | 0.03 |
| Prairies | | | |
| Walleye/Pickerel | 0.21 | 0.01 | 0.42 |
| Northern pike/Jackfish | 0.08 | 0.03 | 0.13 |
| Whitefish | 0.03 | 0.01 | 0.05 |
| Perch | 0.02 | 0.00 | 0.05 |
| Deer kidney | 0.00 | 0.00 | 0.01 |
| Boreal Shield | | | |
| Walleye/Pickerel | 3.02 | 1.90 | 4.14 |
| Northern pike/Jackfish | 1.07 | 0.28 | 1.86 |
| Whitefish | 0.15 | 0.05 | 0.25 |
| Trout | 0.13 | 0.04 | 0.22 |
| Caribou kidney | 0.10 | 0.00 | 0.23 |
| Taiga Shield | | | |
| Caribou kidney | 0.50 | 0.30 | 0.70 |
| Trout | 0.34 | 0.31 | 0.36 |
| Walleye/Pickerel | 0.27 | 0.12 | 0.43 |
| Whitefish | 0.25 | 0.03 | 0.47 |
| Caribou meat | 0.22 | 0.09 | 0.35 |
| Hudson Plains | | | |
| Northern pike/Jackfish | 1.21 | 0.29 | 2.14 |
| Walleye/Pickerel | 1.04 | 0.83 | 1.25 |
| Sturgeon | 0.21 | 0.15 | 0.28 |
| Whitefish | 0.10 | 0.03 | 0.17 |
| Moose meat | 0.04 | 0.02 | 0.05 |
| Mixedwood Plains | | | |
| Walleye/Pickerel | 0.57 | 0.38 | 0.77 |
| Perch | 0.26 | 0.10 | 0.42 |
| Sturgeon | 0.05 | 0.00 | 0.10 |
| Salmon | 0.02 | 0.00 | 0.05 |
| Trout | 0.02 | 0.00 | 0.04 |
| Atlantic Maritime | | | |
| Lobster | 0.19 | 0.16 | 0.22 |
| Atlantic salmon | 0.08 | 0.06 | 0.09 |
| Haddock | 0.05 | 0.03 | 0.07 |
| Halibut | 0.04 | 0.02 | 0.06 |
| Crabs | 0.03 | 0.02 | 0.04 |

**Table 5: Methyl Mercury**

| Sample | Mean (ug/day) | Lower 95% CI (ug/day) | Upper 95% CI (ug/day) |
| --- | --- | --- | --- |
| Pacific Maritime | | | |
| Halibut | 1.46 | 0.63 | 2.30 |
| Rockfish | 0.35 | 0.21 | 0.49 |
| Salmon | 0.16 | 0.10 | 0.23 |
| Cod | 0.07 | 0.00 | 0.13 |
| Prawns | 0.05 | 0.00 | 0.10 |
| Boreal Cordillera | | | |
| Salmon | 0.12 | 0.10 | 0.13 |
| Trout | 0.05 | 0.00 | 0.09 |
| Moose meat | 0.00 | 0.00 | 0.00 |
| Moose liver | 0.00 | 0.00 | 0.00 |
| Montane Cordillera | | | |
| Ling cod/Mariah/Burbot | 0.16 | 0.00 | 0.44 |
| Salmon | 0.11 | 0.07 | 0.15 |
| Trout | 0.07 | 0.02 | 0.12 |
| Halibut | 0.05 | 0.00 | 0.10 |
| Whitefish | 0.01 | 0.01 | 0.01 |
| Taiga Plains | | | |
| Northern pike/Jackfish | 1.06 | 0.30 | 1.81 |
| Walleye/Pickerel | 0.93 | 0.00 | 2.10 |
| Duck meat | 0.03 | 0.00 | 0.06 |
| Salmon | 0.01 | 0.01 | 0.02 |
| Trout | 0.01 | 0.00 | 0.01 |
| Boreal Plains | | | |
| Walleye/Pickerel | 0.50 | 0.25 | 0.75 |
| Northern pike/Jackfish | 0.40 | 0.22 | 0.59 |
| Whitefish | 0.03 | 0.01 | 0.04 |
| Deer meat | 0.01 | 0.00 | 0.02 |
| Trout | 0.01 | 0.00 | 0.02 |
| Prairies | | | |
| Walleye/Pickerel | 0.19 | 0.01 | 0.37 |
| Northern pike/Jackfish | 0.05 | 0.02 | 0.09 |
| Whitefish | 0.02 | 0.01 | 0.04 |
| Perch | 0.02 | 0.00 | 0.05 |
| Duck meat | 0.00 | 0.00 | 0.01 |
| Boreal Shield | | | |
| Walleye/Pickerel | 3.12 | 1.96 | 4.27 |
| Northern pike/Jackfish | 0.66 | 0.17 | 1.15 |
| Trout | 0.12 | 0.04 | 0.19 |
| Whitefish | 0.11 | 0.04 | 0.18 |
| Sturgeon | 0.05 | 0.02 | 0.07 |
| Taiga Shield | | | |
| Trout | 0.41 | 0.38 | 0.45 |
| Walleye/Pickerel | 0.27 | 0.12 | 0.42 |
| Whitefish | 0.24 | 0.03 | 0.45 |
| Caribou meat | 0.19 | 0.08 | 0.30 |
| Northern pike/Jackfish | 0.19 | 0.06 | 0.32 |
| Hudson Plains | | | |
| Northern pike/Jackfish | 0.75 | 0.18 | 1.31 |
| Walleye/Pickerel | 0.65 | 0.52 | 0.78 |
| Sturgeon | 0.15 | 0.10 | 0.20 |
| Whitefish | 0.06 | 0.02 | 0.11 |
| Cisco | 0.02 | 0.01 | 0.03 |
| Mixedwood Plains | | | |
| Walleye/Pickerel | 0.31 | 0.21 | 0.41 |
| Perch | 0.12 | 0.05 | 0.19 |
| Sturgeon | 0.03 | 0.00 | 0.05 |
| Trout | 0.02 | 0.00 | 0.03 |
| Salmon | 0.01 | 0.00 | 0.03 |
| Atlantic Maritime | | | |
| Lobster | 0.14 | 0.12 | 0.17 |
| Atlantic salmon | 0.06 | 0.05 | 0.08 |
| Crabs | 0.03 | 0.02 | 0.04 |
| Shrimp | 0.02 | 0.02 | 0.03 |
| Halibut | 0.02 | 0.01 | 0.03 |

**Table 6: p,p’-DDE**

| Sample | Mean (ng/day) | Lower 95% CI (ng/day) | Upper 95% CI (ng/day) |
| --- | --- | --- | --- |
| Pacific Maritime | | | |
| Ooligan grease | 34.39 | 2.06 | 66.72 |
| Salmon | 14.03 | 8.70 | 19.36 |
| Halibut | 9.83 | 4.21 | 15.44 |
| Salmon eggs | 4.89 | 2.15 | 7.63 |
| Ooligan | 3.14 | 0.71 | 5.57 |
| Boreal Cordillera | | | |
| Salmon | 2.83 | 2.51 | 3.16 |
| Blueberries | 0.00 | 0.00 | 0.00 |
| Trout | 0.00 | 0.00 | 0.00 |
| Moose meat | 0.00 | 0.00 | 0.00 |
| Moose liver | 0.00 | 0.00 | 0.00 |
| Montane Cordillera | | | |
| Salmon | 3.79 | 2.40 | 5.17 |
| Salmon eggs | 3.11 | 0.17 | 6.05 |
| Trout | 2.24 | 0.61 | 3.87 |
| Ooligan grease | 1.48 | 0.00 | 3.97 |
| Ling cod/Mariah/Burbot | 1.24 | 0.00 | 3.37 |
| Taiga Plains | | | |
| Goose meat | 12.93 | 7.84 | 18.03 |
| Duck meat | 2.65 | 0.26 | 5.03 |
| Salmon | 1.22 | 0.55 | 1.88 |
| Northern pike/Jackfish | 0.21 | 0.06 | 0.37 |
| Arctic grayling | 0.06 | 0.00 | 0.12 |
| Boreal Plains | | | |
| Moose meat | 7.94 | 3.07 | 12.81 |
| Moose liver | 2.79 | 0.71 | 4.87 |
| Northern pike/Jackfish | 0.87 | 0.47 | 1.26 |
| Duck meat | 0.45 | 0.17 | 0.73 |
| Whitefish | 0.42 | 0.20 | 0.64 |
| Prairies | | | |
| Deer liver | 2.85 | 0.67 | 5.03 |
| Whitefish | 0.47 | 0.18 | 0.75 |
| Walleye/Pickerel | 0.21 | 0.01 | 0.41 |
| Duck meat | 0.12 | 0.00 | 0.30 |
| Northern pike/Jackfish | 0.03 | 0.01 | 0.05 |
| Boreal Shield | | | |
| Walleye/Pickerel | 10.86 | 6.83 | 14.89 |
| Whitefish | 8.92 | 3.09 | 14.75 |
| Trout | 4.94 | 1.55 | 8.32 |
| Goose meat | 3.70 | 0.58 | 6.83 |
| Ptarmigan meat | 3.66 | 0.00 | 10.70 |
| Taiga Shield | | | |
| Trout | 5.43 | 5.00 | 5.86 |
| Whitefish | 3.41 | 0.37 | 6.45 |
| Duck meat | 2.61 | 1.53 | 3.69 |
| Goose meat | 0.45 | 0.00 | 1.34 |
| Northern pike/Jackfish | 0.17 | 0.05 | 0.29 |
| Hudson Plains | | | |
| Goose meat | 114.44 | 69.54 | 159.34 |
| Northern pike/Jackfish | 2.06 | 0.49 | 3.63 |
| Sturgeon | 1.60 | 1.09 | 2.11 |
| Whitefish | 1.45 | 0.39 | 2.51 |
| Duck meat | 1.00 | 0.57 | 1.43 |
| Mixedwood Plains | | | |
| Salmon | 13.03 | 0.00 | 30.39 |
| Trout | 7.03 | 0.00 | 14.37 |
| Walleye/Pickerel | 5.22 | 3.48 | 6.96 |
| Sturgeon | 2.97 | 0.10 | 5.84 |
| Perch | 1.43 | 0.56 | 2.30 |
| Atlantic Maritime | | | |
| Atlantic salmon | 6.21 | 4.79 | 7.63 |
| Eel | 1.89 | 1.15 | 2.64 |
| Lobster | 1.79 | 1.50 | 2.07 |
| Trout | 1.47 | 1.06 | 1.88 |
| Smelt | 1.10 | 0.68 | 1.53 |

**Table 7: PCBs**

| Sample | Mean (ng/day) | Lower 95% CI (ng/day) | Upper 95% CI (ng/day) |
| --- | --- | --- | --- |
| Pacific Maritime | | | |
| Halibut | 4.77 | 2.04 | 7.49 |
| Pacific herring | 4.06 | 0.00 | 8.63 |
| Salmon | 3.67 | 2.27 | 5.06 |
| Prawns | 2.84 | 0.00 | 5.83 |
| Ooligan grease | 1.69 | 0.10 | 3.28 |
| Boreal Cordillera | | | |
| Blueberries | 0.00 | 0.00 | 0.00 |
| Trout | 0.00 | 0.00 | 0.00 |
| Moose meat | 0.00 | 0.00 | 0.00 |
| Moose liver | 0.00 | 0.00 | 0.00 |
| Black bear fat | 0.00 | 0.00 | 0.00 |
| Montane Cordillera | | | |
| Salmon eggs | 1.74 | 0.10 | 3.39 |
| Salmon | 0.33 | 0.21 | 0.45 |
| Ling cod/Mariah/ Burbot | 0.10 | 0.00 | 0.27 |
| Trout | 0.06 | 0.02 | 0.10 |
| Raspberries | 0.00 | 0.00 | 0.00 |
| Taiga Plains | | | |
| Salmon | 0.37 | 0.17 | 0.58 |
| Trout | 0.05 | 0.03 | 0.07 |
| Northern pike/ Jackfish | 0.00 | 0.00 | 0.00 |
| Walleye/Pickerel | 0.00 | 0.00 | 0.00 |
| Beaver meat | 0.00 | 0.00 | 0.00 |
| Boreal Plains | | | |
| Duck meat | 2.77 | 1.05 | 4.49 |
| Walleye/Pickerel | 0.47 | 0.24 | 0.69 |
| Beaver meat | 0.40 | 0.00 | 0.86 |
| Elk liver | 0.25 | 0.00 | 0.51 |
| Northern pike/ Jackfish | 0.23 | 0.12 | 0.33 |
| Prairies | | | |
| Whitefish | 0.34 | 0.13 | 0.55 |
| Walleye/Pickerel | 0.33 | 0.01 | 0.65 |
| Deer liver | 0.27 | 0.06 | 0.48 |
| Duck meat | 0.03 | 0.00 | 0.07 |
| Perch | 0.00 | 0.00 | 0.00 |
| Boreal Shield | | | |
| Walleye/Pickerel | 45.21 | 28.43 | 61.99 |
| Ptarmigan meat | 24.51 | 0.00 | 71.72 |
| Whitefish | 21.33 | 7.39 | 35.27 |
| Duck meat | 20.72 | 0.00 | 45.47 |
| Trout | 12.09 | 3.81 | 20.37 |
| Taiga Shield | | | |
| Black bear fat | 16.29 | 0.00 | 56.04 |
| Trout | 6.08 | 5.60 | 6.56 |
| Whitefish | 5.26 | 0.57 | 9.96 |
| Duck meat | 3.27 | 1.92 | 4.62 |
| Northern pike/ Jackfish | 0.22 | 0.07 | 0.37 |
| Hudson Plains | | | |
| Northern pike/ Jackfish | 4.19 | 1.00 | 7.39 |
| Goose meat | 2.74 | 1.66 | 3.81 |
| Sturgeon | 1.90 | 1.30 | 2.51 |
| Whitefish | 1.80 | 0.49 | 3.12 |
| Walleye/Pickerel | 1.77 | 1.41 | 2.13 |
| Mixedwood Plains | | | |
| Sturgeon | 43.16 | 1.40 | 84.92 |
| Salmon | 37.53 | 0.00 | 87.49 |
| Walleye/Pickerel | 36.18 | 24.09 | 48.27 |
| Trout | 19.25 | 0.00 | 39.34 |
| Catfish | 12.06 | 0.00 | 31.76 |
| Atlantic Maritime | | | |
| Atlantic salmon | 7.49 | 5.78 | 9.21 |
| Mackerel | 1.90 | 0.84 | 2.96 |
| Trout | 1.78 | 1.28 | 2.27 |
| Eel | 1.77 | 1.07 | 2.46 |
| Lobster | 1.39 | 1.17 | 1.61 |
