# Supplementary material for: Levels of metals and persistent organic pollutants in traditional foods consumed by First Nations living on-reserve in Canada
Source: Can J Public Health. 2021 Jun 28;112(Suppl 1):81–96. doi: 10.17269/s41997-021-00495-7 (PMC8239065; doi:10.17269/s41997-021-00495-7)
Supplement: Supplementary file 5 — (DOCX 12 kb) [file 41997_2021_495_MOESM5_ESM.docx]

**Supplementary Material E**

A summary of the body weight of the participants

| **Body Weight (Consumers of Traditional Foods)** | | | | |
| --- | --- | --- | --- | --- |
|  | **N** | **Mean ± SD (kg)** | **Median (kg)** | **Range (kg)** |
| **Total** | 6105 | 84.60 ± 19.08 | 81.82 | 32.09 – 221.36 |
| **Females** | 4003 | 81.21 ± 17.97 | 79.45 | 32.09 – 169.54 |
| **Males** | 2102 | 91.05 ± 19.47 | 88.64 | 30.09 – 221.36 |
