# Supplementary material for: Levels of metals and persistent organic pollutants in traditional foods consumed by First Nations living on-reserve in Canada
Source: Can J Public Health. 2021 Jun 28;112(Suppl 1):81–96. doi: 10.17269/s41997-021-00495-7 (PMC8239065; doi:10.17269/s41997-021-00495-7)
Supplement: Supplementary file 6 — (DOCX 23 kb) [file 41997_2021_495_MOESM6_ESM.docx]

Supplementary Material F.

Concentrations of total mercury and methylmercury in traditional samples collected.

| **Sample** | **Total Hg**  **N_sites_** | **MeHg N_sites_** | **Hg: Mean ± SD (µg/g)** | **MeHg: Mean ± SD (µg/g)** | **MeHg/Hg**  **(%)** |
| --- | --- | --- | --- | --- | --- |
| Seaweed | 5 | 2 | ND | ND | NA |
| Carp | 2 | 2 | 0.5425 ± 0.2468 | 0.1600 ± 0.0311 | 29.5 |
| Eel | 10 | 9 | 0.1260 ± 0.0478 | 0.1103 ± 0.0464 | 87.5 |
| Halibut | 9 | 8 | 0.1729 ± 0.1047 | 0.2083 ± 0.1106 | 120.5 |
| Atlantic Herring | 2 | 2 | 0.1001 ± 0.0182 | 0.0708 ± 0.0331 | 70.7 |
| Mackerel | 8 | 8 | 0.0319 ± 0.0080 | 0.0237 ± 0.0111 | 74.4 |
| Perch | 11 | 9 | 0.1773 ± 0.0767 | 0.0978 ± 0.0469 | 55.2 |
| Northern Pike or Jackfish | 37 | 34 | 0.4352 ± 0.4703 | 0.2715 ± 0.1965 | 62.4 |
| Rockfish | 6 | 6 | 0.1670 ± 0.1280 | 0.2425 ± 0.1255 | 145.2 |
| Shad | 1 | 1 | 0.0769 | 0.0277 | 36.0 |
| Smelt | 15 | 13 | 0.0405 ± 0.0259 | 0.0271 ± 0.0209 | 66.9 |
| Sturgeon | 13 | 10 | 0.2379 ± 0.1908 | 0.1816 ± 0.1543 | 76.4 |
| Northern Abalone | 1 | 1 | ND | ND | NA |
| Clams | 13 | 8 | 0.0075 ± 0.0078 | 0.0040 ± 0.0046 | 53.6 |
| Mussels | 6 | 3 | 0.0220 ± 0.0220 | 0.0090 ± 0.0021 | 41.2 |
| Squid | 2 | 2 | 0.0304 ± 0.0016 | 0.0341 ± 0.0005 | 112.2 |
| Bass | 11 | 9 | 0.4045 ± 0.2989 | 0.3253 ± 0.4603 | 80.4 |
| Striped Bass | 7 | 6 | 0.1551 ± 0.0915 | 0.1268 ± 0.0991 | 81.8 |
| Ling Cod or Mariah or Burbot | 6 | 4 | 0.2113 ± 0.1290 | 0.2358 ± 0.1462 | 111.6 |
| Pacific Herring | 1 | 1 | 0.0160 | 0.0260 | 162.5 |
| Walleye or Pickerel | 49 | 41 | 0.3822 ± 0.2496 | 0.2983 ± 0.3146 | 78.1 |
| Cod | 8 | 7 | 0.0822 ± 0.0903 | 0.0629 ± 0.0544 | 76.5 |
| Atlantic Salmon | 17 | 17 | 0.0643 ± 0.0365 | 0.0566 ± 0.0333 | 88.0 |
| Sucker | 15 | 12 | 0.0716 ± 0.0676 | 0.0751 ± 0.0606 | 105.0 |
| Whitefish | 43 | 37 | 0.0954 ± 0.1008 | 0.0636 ± 0.0578 | 66.6 |
| Crabs | 14 | 8 | 0.0786 ± 0.0512 | 0.0701 ± 0.0557 | 89.2 |
| Octopus | 1 | 1 | 0.0370 | 0.0430 | 116.2 |
| Oysters | 4 | 1 | 0.0161 ± 0.0056 | 0.0050 | 31.2 |
| Catfish | 6 | 6 | 0.1293 ± 0.0854 | 0.0918 ± 0.0355 | 71.0 |
| Haddock | 2 | 2 | 0.0449 ± 0.0069 | 0.0211 ± 0.0067 | 46.9 |
| Lobster | 12 | 10 | 0.1269 ± 0.0923 | 0.1246 ± 0.1329 | 98.2 |
| Shrimp | 2 | 2 | 0.0324 ± 0.0072 | 0.0324 ± 0.0187 | 100.0 |
| Trout | 82 | 74 | 0.1919 ± 0.1940 | 0.1886 ± 0.2018 | 98.3 |
| Beaver Meat | 29 | 9 | 0.0011 0.0036 | 0.0001 ± 0.0004 | 12.6 |
| Bison Meat | 5 | 1 | 0.0007 ± 0.0016 | ND | 0.0 |
| Caribou Meat | 20 | 3 | 0.0102 ± 0.0072 | 0.0076 ± 0.0072 | 74.9 |
| Deer Meat | 65 | 11 | 0.0015 ± 0.0029 | 0.0009 ± 0.0020 | 63.2 |
| Elk Meat | 24 | 4 | 0.0002 ± 0.0007 | ND | 0.0 |
| Goat Meat | 2 | 2 | ND | ND | NA |
| Moose Meat | 105 | 21 | 0.0018 ± 0.0044 | 0.0002 ± 0.0010 | 12.2 |
| Rabbit or Hare Meat | 58 | 9 | 0.0021 ± 0.0047 | ND | 0.0 |
| Harp Seal Meat | 1 | 1 | 1.0600 | 1.3900 | 131.1 |
| Goose Meat | 39 | 18 | 0.0015 ± 0.0013 | 0.0009 ± 0.0011 | 55.7 |
| Scallops | 9 | 6 | 0.0176 ± 0.0160 | 0.0175 ± 0.0187 | 99.8 |
| Sea Snails | 1 | 1 | 0.0212 | 0.0162 | 76.4 |
| Caribou Brain | 3 | 1 | 0.0023 ± 0.0004 | ND | 0.0 |
| Caribou Heart | 5 | 1 | 0.0059 ± 0.0010 | 0.0043 | 73.1 |
| Moose Kidney | 40 | 16 | 0.0156 ± 0.0124 | ND | 0.0 |
| Moose Liver | 49 | 18 | 0.0056 ± 0.0083 | 0.0007 ± 0.0017 | 12.8 |
| Black Bear Meat | 15 | 3 | 0.0036 ± 0.0034 | 0.0011 ± 0.0020 | 31.8 |
| Arctic Char | 1 | 1 | 0.9190 | 0.7360 | 80.1 |
| Arctic Grayling | 2 | 1 | 0.0950 ± 0.1018 | 0.0347 | 36.5 |
| Herring Eggs | 6 | 6 | ND | ND | NA |
| Ling Cod or Mariah Liver | 2 | 2 | 0.0200 ± 0.0071 | 0.0444 ± 0.0226 | 222.0 |
| Cisco | 4 | 2 | 0.0652 ± 0.0259 | 0.0258 ± 0.0177 | 39.6 |
| Duck Meat | 73 | 22 | 0.0482 ± 0.0640 | 0.0642 ± 0.0891 | 133.2 |
| Grouse Meat | 82 | 7 | 0.0007 ± 0.0011 | 0.0006 ± 0.0017 | 91.9 |
| Sea Cucumber | 1 | 1 | 0.0050 | ND | 0.0 |
| Muskrat Meat | 10 | 4 | 0.0020 ± 0.0026 | 0.0016 ± 0.0020 | 80.4 |
| Moose Heart | 28 | 4 | 0.0013 ± 0.0019 | ND | 0.0 |
| Elk Kidney | 3 | 1 | 0.0089 ± 0.0155 | ND | 0.0 |
| Elk Liver | 2 | 2 | ND | ND | NA |
| Beaver Heart | 1 | 1 | ND | ND | NA |
| Beaver Kidney | 1 | 1 | 0.0070 | ND | 0.0 |
| Beaver Liver | 2 | 1 | 0.0011 ± 0.0016 | ND | 0.0 |
| Deer Liver | 18 | 7 | 0.0107 ± 0.0155 | 0.0020 ± 0.0036 | 19.0 |
| Deer Heart | 4 | 2 | 0.0087 ± 0.0156 | ND | 0.0 |
| Bear Liver | 1 | 1 | ND | 0.0070 | NA |
| Deer Kidney | 9 | 3 | 0.0360 ± 0.0450 | ND | 0.0 |
| Caribou Liver | 3 | 1 | 0.1342 ± 0.1140 | 0.0095 | 7.1 |
| Caribou Kidney | 4 | 1 | 0.5902 ± 0.4025 | ND | 0.0 |
| Rabbit or Hare Liver | 5 | 1 | 0.0106 ± 0.0046 | ND | 0.0 |
| Rabbit or Hare Kidney | 2 | 1 | 0.0327 ± 0.0122 | ND | 0.0 |
| Elk Heart | 1 | 1 | ND | ND | NA |
| Black Bear Fat | 8 | 1 | 0.0018 ± 0.0051 | ND | 0.0 |
| Salmon | 57 | 53 | 0.0425 ± 0.0576 | 0.0501 ± 0.0593 | 117.9 |
| Ooligan | 4 | 4 | 0.0110 ± 0.0051 | 0.0155 ± 0.0045 | 140.9 |
| Mooneye or Goldeye | 2 | 1 | 0.1381 ± 0.0904 | 0.0805 | 58.3 |
| Sole | 2 | 2 | 0.0517 ± 0.0486 | 0.0494 ± 0.0496 | 95.6 |
| Flounder | 2 | 2 | 0.0455 ± 0.0300 | 0.0337 ± 0.0310 | 74.0 |
| Salmon Eggs | 11 | 11 | 0.0361 ± 0.0584 | 0.0014 ± 0.0026 | 3.9 |
| Trout Eggs | 3 | 3 | 0.0050 ± 0.0027 | 0.0032 ± 0.0037 | 62.9 |
| Sucker Eggs | 3 | 1 | 0.0096 ± 0.0029 | 0.0028 | 29.3 |
| Walleye or Pickerel Eggs | 1 | 1 | 0.0074 | 0.0026 | 35.1 |
| Northern Pike or Jackfish Eggs | 2 | 2 | 0.0299 ± 0.0327 | 0.0224 ± 0.0316 | 74.7 |
| Ooligan Grease | 5 | 3 | 0.0006 ± 0.0013 | 0.0020 ± 0.0035 | 333.3 |
| Walleye or Pickerel Pemmican | 1 | 1 | 0.2120 | 0.0739 | 34.9 |
| Cockles | 3 | 3 | 0.0507 ± 0.0835 | 0.0490 ± 0.0797 | 96.7 |
| Prawns | 3 | 3 | 0.0110 ± 0.0096 | 0.0227 ± 0.0065 | 206.1 |
| Arctic Tern or Stern Egg | 1 | 1 | 0.0549 | 0.0354 | 64.5 |
| Duck Gizzard | 5 | 2 | 0.0364 ± 0.0263 | 0.0624 ± 0.0426 | 171.5 |
| Goose Kidney | 1 | 1 | 0.0010 | ND | 0.0 |
| Goose Liver | 1 | 1 | 0.0150 | 0.0103 | 68.7 |
| Duck Liver | 1 | 1 | 0.1170 | 0.1420 | 121.4 |
| Goose Gizzard | 2 | 1 | 0.0020 ± 0.0028 | ND | 0.0 |
| Whitefish Eggs | 2 | 2 | 0.0108 ± 0.0153 | 0.0034 ± 0.0047 | 31.0 |
| Deer Liver and Heart | 2 | 1 | 0.0121 ± 0.0069 | 0.0060 | 49.6 |
